# The Evolutionary Conserved γ-Core Motif Influences the Anti-*Candida* Activity of the *Penicillium chrysogenum* Antifungal Protein PAF

**DOI:** 10.3389/fmicb.2018.01655

**Published:** 2018-07-20

**Authors:** Christoph Sonderegger, Györgyi Váradi, László Galgóczy, Sándor Kocsubé, Wilfried Posch, Attila Borics, Sandrine Dubrac, Gábor K. Tóth, Doris Wilflingseder, Florentine Marx

**Affiliations:** ^1^Biocenter, Division of Molecular Biology, Innsbruck Medical University, Innsbruck, Austria; ^2^Department of Medical Chemistry, Faculty of Medicine, University of Szeged, Szeged, Hungary; ^3^Institute of Plant Biology, Biological Research Centre, Hungarian Academy of Sciences, Szeged, Hungary; ^4^Department of Microbiology, Faculty of Science and Informatics, University of Szeged, Szeged, Hungary; ^5^Division of Hygiene and Medical Microbiology, Innsbruck Medical University, Innsbruck, Austria; ^6^Institute of Biochemistry, Biological Research Centre, Hungarian Academy of Sciences, Szeged, Hungary; ^7^Department of Dermatology, Venereology and Allergy, Innsbruck Medical University, Innsbruck, Austria; ^8^MTA-SZTE Biomimetic Systems Research Group, University of Szeged, Szeged, Hungary

**Keywords:** *Penicillium chrysogenum* antifungal protein PAF, gamma(γ-) core, antimicrobial proteins, antimicrobial peptides, *Candida albicans*, biofilm, reactive oxygen species

## Abstract

Small, cysteine-rich and cationic antimicrobial proteins (AMPs) from filamentous ascomycetes represent ideal bio-molecules for the development of next-generation antifungal therapeutics. They are promising candidates to counteract resistance development and may complement or even replace current small molecule-based antibiotics in the future. In this study, we show that a 14 amino acid (aa) long peptide (Pγ) spanning the highly conserved γ-core motif of the *Penicillium chrysogenum* antifungal protein (PAF) has antifungal activity against the opportunistic human pathogenic yeast *Candida albicans*. By substituting specific aa we elevated the positive net charge and the hydrophilicity of Pγ and created the peptide variants Pγ^var^ and Pγ^opt^ with 10-fold higher antifungal activity than Pγ. Similarly, the antifungal efficacy of the PAF protein could be significantly improved by exchanging the respective aa in the γ-core of the protein by creating the protein variants PAFγ^var^ and PAFγ^opt^. The designed peptides and proteins were investigated in detail for their physicochemical features and mode of action, and were tested for cytotoxicity on mammalian cells. This study proves for the first time the important role of the γ-core motif in the biological function of an AMP from ascomycetes. Furthermore, we provide a detailed phylogenetic analysis that proves the presence and conservation of the γ-core motif in all AMP classes from Eurotiomycetes. We emphasize the potential of this common protein motif for the design of short antifungal peptides and as a protein motif in which targeted aa substitutions enhance antimicrobial activity.

## Introduction

Fungal pathogens have a severe impact on public health. Among all opportunistic human pathogenic fungi, *Candida* species are the most common, whereby *Candida albicans* is the major representative (Brown et al., 2012). *Candida* spp. can cause life-threatening invasive bloodstream infections (fungemia) with mortality rates as high as 75%, but also superficial or mucosal infections (Brown et al., 2012; Duncan and O’Neil, 2013). Whereas fungemia mostly has a fatal outcome, superficial infections are not life-threatening, but appear with higher frequency affecting approx. 25% of the world population (Brown et al., 2012). Although superficial infections are mostly easy to cure, treatment may get more challenging in the future, since *Candida* species are known to have either intrinsic resistance or the potential of resistance formation to conventional antifungal drugs (Brown et al., 2012; Rautenbach et al., 2016; Sanglard, 2016). Some reports suggest that we are already on the brink of a “post-antibiotic era” as a consequence of the emerging number of resistant strains and limited number of effective antifungal agents (Kosikowska and Lesner, 2016; Kang et al., 2017). To overcome this problem, the development of new antifungal strategies is urgently needed. One possible solution to replace conventional antibiotics is offered by antimicrobial peptides and proteins (AMPs) that are produced, for example, by filamentous ascomycetes (Hegedüs and Marx, 2013; Garrigues et al., 2016; Tóth et al., 2016). One of the most extensively characterized AMPs from this class is the *Penicillium chrysogenum* antifungal protein (PAF), a 55 amino acid (aa) long, cysteine-rich, and cationic protein secreted by the β-lactam antibiotic producer *P. chrysogenum.* PAF shows potent growth inhibitory activity against numerous filamentous plant- and human-pathogenic fungi (Marx et al., 2008; Hegedüs et al., 2011), and only recently we also reported on its anti-*Candida* effect (Huber et al., 2018).

**FIGURE 1:**
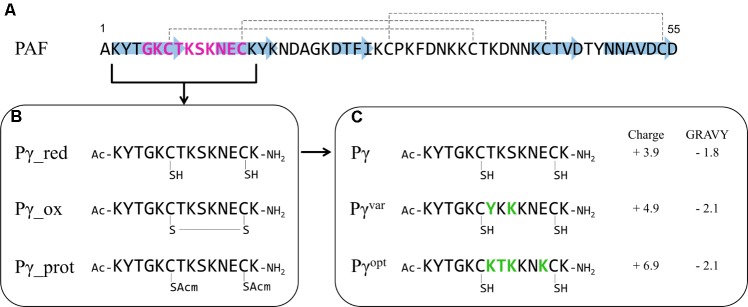
Synthetic PAF γ-core peptides. **(A)** The primary structure of PAF with indicated β-strands (blue arrows) and disulfide bonds (dashed lines). The γ-core motif is highlighted in purple. **(B)** Pγ peptide spanning the γ-core motif of PAF in different modifications: red – Cys in reduced form (–SH), ox – cyclic peptide with closed disulfide bonds (S–S), prot – Cys with a protecting group (SAcm). The sulfur (S) atom of the Cys is indicated. The N- and C-termini of the Pγ peptides are acetylated (Ac) and amidated (NH_2_), respectively. **(C)** The reduced Pγ form and the derived peptide variants Pγ^var^ and Pγ^opt^. Amino acid substitutions are highlighted in green, the net charge (at pH 7, http://protcalc.sourceforge.net) and GRAVY (www.gravy-calculator.de) are indicated.

PAF consists of five antiparallel β-strands that are connected by four flexible loops and is stabilized to a compact β-structure by three intramolecular disulfide bonds (**Figure 1A**; Batta et al., 2009; Fizil et al., 2015). A 10 aa long sequence located in loop 1 at the N-terminus resembles the structural conserved gamma(γ)-core motif of cysteine-stabilized small proteins. The γ-core motif consists of the consensus aa sequence GXCX_3-9_C that can be found virtually in all classes of cysteine-stabilized AMPs from prokaryotes and eukaryotes (Yount and Yeaman, 2006). This motif can either be present in its dextromeric isoform (X_1-3_-GXC-X_3-9_-C) or in one of its levomeric isoforms (C-X_3-9_-CXG-X_1-3_ or C-X_3-9_-GXC-X_1-3_) (Yount and Yeaman, 2004; Yeaman and Yount, 2007). The γ-core is not only conserved in its aa sequence, but also in its structure. It consists of two antiparallel β-strands, connected by an interposed short turn region (Yount and Yeaman, 2004). This “core structure” can either function as a stand-alone molecule as in the case of antimicrobial protegrins, or it can be the scaffold for modular constructed proteins where β-sheets and/or α-helices are attached to the γ-core (Yount and Yeaman, 2004). Apart from AMPs this motif can also be found in a huge variety of host defense polypeptides such as thionins, venoms, toxins, and kinocidins (Yeaman and Yount, 2007). The observation that γ-core containing peptide classes exert direct antimicrobial function renders the γ-core a worthwhile subject for antimicrobial investigations. Indeed, this motif was already found to be an important functional determinant of antifungal plant defensins and deduced γ-core peptide fragments showed antifungal activity (Sagaram et al., 2011). Recent studies with the *Neosartorya fischeri* antifungal protein NFAP2 and the *Penicillium digitatum* antifungal protein AfpB, however, revealed that the dextromeric γ-core motif does not influence the growth inhibitory activity of these ascomycetous AMPs (Garrigues et al., 2017; Tóth et al., 2018).

In this study, we report for the first time the significant influence of the dextromeric γ-core motif of the *P. chrysogenum* PAF on its mode of action. We show that a synthetic 14 aa long peptide fragment (Pγ) spanning the PAF γ-core motif exhibits *in vitro* antifungal activity against *C. albicans.* Specific aa exchanges that elevate the positive net charge and hydrophilicity of Pγ resulted in significantly improved antifungal activity of the respective peptide variants Pγ^var^ and Pγ^opt^. The same aa exchanges in the γ-core motif of the full-length protein comparably enhanced the antifungal efficacy of the respective PAF variants, PAF^var^ and PAF^opt^. The functionally improved peptide and protein variants were thoroughly characterized for their physicochemical properties, anti-*Candida* mode of action, and cytotoxic potential against mammalian cells *in vitro*. Since the evolutionary conserved γ-core motif is present in phylogenetically distinct AMP classes from Eurotiomycetes, our PAF-based results foster further investigations of the structure–function relation of related AMPs and promise the development of proteins and peptides with increased antimicrobial efficacy by rational design.

## Materials and Methods

### Peptide Synthesis

Peptides were synthesized on solid phase applying Fmoc chemistry and dicyclohexylcarbodiimide/1-hydroxy-benzotriazole (DCC/HOBt) coupling with a threefold excess of reagents. PL-Rink Resin (Varian, Inc.) or Rink-amide-MBHA resin (SENN Chemicals) was used as a solid support. In case of Pγ_prot, cysteines (Cys) were incorporated as Fmoc-Cys(Acm)-OH. After completion of the synthesis, the N-terminus of each peptide was acetylated. Peptides were detached from the resin with a 95:5 (v/v) trifluoroacetic acid (TFA)/water mixture containing 3% (w/v) dithiothreitol (DTT) at room temperature (RT) for 2.5 h. The resin was removed and the peptides were precipitated by the addition of ice cold diethyl ether.

To form Pγ_ox, sulfhydryl groups of Cys of 40 mg crude Pγ_red were oxidized to disulfide bonds by atmospheric oxygen in a 0.1 M ammonium acetate buffer (pH 7.5) applying intensive stirring in an open bottle at RT for 48 h.

Peptides were purified by preparative RP-HPLC using a C18 column with a solvent system of (A) 0.1% (v/v) TFA in water and (B) 80% (v/v) acetonitrile and 0.1% TFA (v/v) in water at a flow rate of 4.0 mL min^-1^. The absorbance was detected at 220 nm. The appropriate fractions were pooled and lyophilized.

Purity of the peptides was characterized by analytical RP-HPLC at a flow rate of 1.0 mL min^-1^. The identity of the peptides was verified by ESI-MS.

### Strains and Growth Conditions

Fungal strains used in this study are listed in Supplementary Table S1 and composition of growth media is given in Supplementary Table S2. All *P. chrysogenum* shaking cultures were inoculated with 2 × 10^8^ conidia, in 200 mL defined minimal medium (*Pc*MM) for protein expression or complete medium (CM) before transformation, and grown at 25°C as described (Sonderegger et al., 2016). For conidial suspension generation from surface cultures, *P. chrysogenum* was grown on *Pc*MM agar at 25°C. *C. albicans* was used as PAF-sensitive model organism and was cultivated on yeast extract peptone dextrose (YPD) agar at 37°C. For growth inhibition and susceptibility assays, *C. albicans* was grown in 20-fold diluted potato dextrose broth (PDB, Sigma-Aldrich) at 30°C, pH 5.5 (Huber et al., 2018).

### Generation of Recombinant Proteins

PAF and its γ-core variants were generated and expressed as described earlier (Sonderegger et al., 2016). Oligonucleotides for PCR-based *paf* gene mutation are listed in Supplementary Table S3. In brief, for vector construction of pSK257*paf*^T8Y S10K^ by site-directed mutagenesis the primer pairs M13/opaf^T8Y S10K_fw^ and opaf12/opaf^T8Y S10K_rev^ were applied on the plasmid pSK257*paf* for the first PCR reactions and T7var/opaf11 were then used to combine the resulting fragments. For the construction of pSK257*paf*^T8KK9TS10KE13K^ the primer pairs opaf15/opaf^T8KK9TS10KE13K_fw^, opaf14/opaf^T8KK9TS10KE13K_rev^, and M13/opaf11 were used, respectively.

The correct vector assembly and nucleotide sequences were verified with Sanger sequencing (Eurofins Genomics, Germany) using opaf10 as a sequencing primer. After vector transfection into *P. chrysogenum*Δ*paf* and protein expression, the purification was performed as described before by cation exchange chromatography (Sonderegger et al., 2016). The purity of the proteins was checked by sodium dodecyl sulfate polyacrylamide gel electrophoresis (SDS–PAGE) with subsequent Coomassie or silver staining and the protein identity and correct processing were verified by ESI-MS at the Protein Micro-Analysis Facility (Medical University of Innsbruck) (Sonderegger et al., 2016).

### Electronic Circular Dichroism (ECD) Spectroscopy

ECD spectroscopic measurements were performed in the far-UV wavelength range (185–260 nm) using a Jasco J-815 spectropolarimeter (JASCO Corporation). Protein samples, dissolved in pure H_2_O in 0.1 mg mL^-1^ concentrations, were measured in a 0.1 cm path length quartz cuvette. Initially, ECD spectra of samples were recorded at 25°C with a scan speed of 100 nm s^-1^. Then, the temperature was increased gradually up to 95°C at 1°C min^-1^ rate, using a Peltier thermoelectric controller (TE Technology). The system was allowed to equilibrate for 1 min before measurements were taken at each temperature point. Changes in the ellipticity at 228 nm (appointed by the spectra measured at 25°C) were recorded during gradual heating and then plotted as a function of temperature to provide thermal unfolding curves of the proteins. The unfolding curves were fitted with an inverse sigmoidal function of which inflexion point designated the melting temperature (*T*_m_) of the native protein structures. ECD spectra were also recorded at 95°C, the final temperature of the thermal unfolding experiments, and then samples were allowed to cool and measured again after 5 min of equilibration at 25°C. The reported spectra are accumulations of 10 scans, from which the corresponding solvent spectrum, recorded similarly, was subtracted. Data are given in mdeg units. Deconvolution analysis of the resultant spectra was performed using the CDSSTR method (Sreerama and Woody, 2000).

### Antifungal Activity Determination

The MIC of all protein and peptide variants was determined on *C. albicans* with broth microdilution assays in 96-well microtiter plates (Nunclon Delta, Thermo Fisher Scientific). From a fresh *C. albicans* overnight culture on YPD agar, cells were harvested with an inoculation loop and suspended in 0.05× PDB (Sigma-Aldrich) (Arendrup et al., 2016). The cell number was determined with a counting chamber (Bürker) and the cell number was adjusted to 10^4^ mL^-1^. In each well, 10^3^ cells were incubated with increasing concentrations of antifungal proteins or peptides in a final volume of 200 μL 0.05× PDB. The plates were incubated for 24 h at 30°C before the growth was determined by measuring the optical density (OD_620nm_) with a multi-mode microplate reader (FLUOstar Omega, BMG Labtech). The MIC was defined as the protein concentration that inhibited growth by ≥90% compared to the untreated control, which was referred as 100% growth.

Fungistatic or fungicidal activities were determined by incubating *C. albicans* (10^4^ cells mL^-1^) in 0.05× PDB with proteins and peptides at 1× MIC and 2× MIC of at 30°C under continuous shaking (800 rpm) in a Thermomixer Comfort (Eppendorf). At defined time points, aliquots were taken and appropriate dilutions were plated on YPD agar in duplicates to promote growth of viable cells. Agar plates were incubated at 37°C for 24 h to count colony forming units (cfu) (Huber et al., 2018).

The antifungal activity against *C. albicans* biofilms was determined in 96-well microtiter plates (Nunclon Delta, Thermo Fisher Scientific). Cells (10^5^) in 100 μL 0.05× PDB were distributed per well and plates were incubated at 37°C for up to 24 h to allow biofilm formation. After cell adherence, the microtiter plates were washed once with 0.05× PDB and the test substances were added in increasing concentrations in 100 μL 0.05× PDB per well. H_2_O_2_ served as a control for cell killing. The plates were further incubated at 30°C for 24 h. The wells were washed once with 0.05× PDB and 100 μL of FDA solution (40 μg mL^-1^ in 0.05× PDB) was added to the wells. Wells containing only FDA solution (40 μg mL^-1^ in 0.05× PDB) were used as blank background control. The plates were incubated for 1 h at 37°C in the dark before the fluorescence signal intensity was measured in a multi-mode microplate reader (FLUOstar Omega, BMG Labtech) with excitation and emission wavelengths of 485 and 520 nm. All experiments were prepared at least in duplicates and repeated at least twice.

### Flow Cytometry

To analyze changes in cell size and to assess kinetics of cell death of *C. albicans* upon antifungal treatment flow cytometry was applied. For both experiments, 10^4^ cells mL^-1^ was incubated in 0.05× PDB with the desired AMP concentration for different time points. The incubation was performed in 1.5 mL reaction tubes at 30°C under continuous shaking at 800 rpm in a Thermomixer Comfort (Eppendorf). After incubation, the cells were centrifuged at 4°C with 3,000 ×*g* for 3 min. For cell size determination, the supernatant was removed and the cells were resuspended and fixed in PBS containing 3.7% (v/v) formaldehyde before changes in cell size were measured by plotting SSC against FSC. *C. albicans* that were incubated at 95°C for 120 min served as controls. Microscopic control-images were taken with a fluorescence microscope (Axioplan, Zeiss), equipped with a camera (Axiocam 503 mono, Zeiss) and excitation/emission filters (365/420 nm for blue fluorescence and 546/590 or 565/620 nm for red fluorescence, Zeiss). Image acquisition was performed with ZEN 2 microscope software (Zeiss, blue edition) and the cell area was measured with ImageJ 1.52d software (Schindelin et al., 2012).

For dead-cell staining, prior to fixation, the cells were incubated in the dark for 30 min at 4°C with 100 μL PBS containing 0.1 μL LIVE/DEAD^TM^ Fixable Far Red Dead Cell Stain (Thermo Fisher Scientific). The cells were then washed once in PBS and fixed in PBS containing 3.7% formaldehyde. Ethanol-inactivated dead *C. albicans* served as a positive staining control. The samples were then analyzed by FACS (FACSVerse Flow Cytometer, BD Biosciences), equipped with BD FACSuite software (v1.0.6.5230, BD Biosciences). Dead cells were detected at Excitation/Emission wavelength of 650/665 nm. Stained, live, and dead *Candida* cells were used as controls to discriminate populations and to set the gates.

### Production of Reactive Oxygen Species (ROS)

For the detection of ROS generation in *C. albicans* a dichloro-dihydro-fluorescein diacetate (DCFH-DA, Sigma-Aldrich)-based fluorometric assay was applied, adapted from Han et al. (2016). Cells from an overnight culture of *C. albicans*, grown on YPD agar, were suspended in 0.05× PDB (Sigma-Aldrich) and the cell number was adjusted to 10^7^ cells mL^-1^. DCFH-DA stock solution (100 mM in DMSO) was added to the cell suspension to give a final concentration of 100 μM. This cell suspension was pre-incubated for 30 min at 30°C protected from light. Serial dilutions of the test compounds were prepared in 0.05× PDB (Sigma-Aldrich) in 96-well microtiter plates (Nunclon Delta, Thermo Fisher Scientific) in a volume of 100 μL per well. H_2_O_2_ served as a positive control for ROS induction. After pre-incubation, the DCFH-DA loaded cells were added to the test compounds (100 μL per well) to give a final volume of 200 μL in each well and the 96-well plate was placed in a multi-mode microplate reader (FLUOstar Omega, BMG Labtech), set to 30°C incubation temperature. The fluorescence signal was recorded every 5 min over a time course of 4 h at excitation and emission wavelengths of 485 and 520 nm, respectively. Measurements were prepared in duplicates and assays were performed at least three times.

### Cytotoxicity Assessment

The hemolytic potential of the protein and peptide variants was tested on Columbia blood agar plates (VWR). Sterile filter discs (Ø6 mm) were placed on the agar plates and loaded with 20 μg (∼10 μL) of each protein and peptide. Sterile water and 20% Triton-X 100 served as negative and positive control, respectively. The plates where incubated for 24 h at 37°C before evaluation.

AMP toxicity was determined on HKC and HDF. Keratinocytes and fibroblasts were isolated and grown in CnT-07 (CellnTEC) or R10 (Supplementary Table S2), respectively, as described previously (Blunder et al., 2017). Fluorescence viability staining was performed on HKC and HDF cells grown on chambered cell culture slides (Falcon). 4 × 10^3^ cells per well were seeded and grown at 37°C and 5% CO_2_ before AMPs were added at 30 μM for 24 h. Cells were washed with PBS and the fluorescent dyes PI (1 μg mL^-1^) and Hoechst 33342 (20 μg mL^-1^) were added for 10 min in the dark. The cells were washed three times with PBS and observed with a Zeiss Axioplan fluorescence microscope (Zeiss), equipped with an Axiocam 503 mono microscope camera (Zeiss), excitation/emission filters 365/420 nm for blue fluorescence, and 546/590 or 565/620 nm for red fluorescence. Image acquisition and editing were done with ZEN 2 (blue edition) microscope software (Zeiss) and GNU Image Manipulation Program (GIMP 2, version 2.8.10).

### Screening for Presence of γ-Core Motifs in AMPs and Phylogenetic Analysis

All genomes from Eurotiomycetes available in MycoCosm portal (Grigoriev et al., 2014) of DOE Joint Genome Institute (JGI) were screened for the presence of γ-core motif-containing AMPs. Full-length sequence of *P. chrysogenum* PAF (Acc. No.: Q01701), *Aspergillus giganteus* AFP (Acc. No.: P17737), *P. brevicompactum* “bubble protein” (BP, Acc. No.: G5DC88), and *N. fischeri* NFAP2 (Acc. No.: A0A1D0CRT2) from the Universal Protein Resource database (UniProt Consortium, 2017) were used as templates for similarity searches applying the Basic Local Alignment Search Tool (BLAST) (Pevsner, 2009). The applied templates covered all recently known groups of AMPs (Garrigues et al., 2016; Tóth et al., 2016). From the same species or isolates only those hits were retained which differed at least in one aa in their sequence. The cleavage site of the signal sequence was predicted with SignalP 4.1 server (Nielsen, 2017). ClustalW multiple alignments were generated with the BioEdit program (Hall, 1999) and visualized with Jalview version 2.10.3b1 (Waterhouse et al., 2009). The grand average of hydropathy (GRAVY) value and total net charge of γ-cores were calculated with GRAVY Calculator^1^ and Protein Calculator v3.4^2^ (The Scripps Research Institute) servers, respectively.

The alignment of the full-length proteins was generated with PRANK v. 140110 (Löytynoja and Goldman, 2010) with default settings for phylogenetic analysis. Phylogenetic information of indels were coded by using FastGap v. 1.2 (Borchsenius, 2009) implementing the simple gap coding algorithm. Maximum-likelihood analysis was performed with RAxML v. 8.2.10 (Stamatakis, 2014) under the PROTGAMMA model with WAG substitution matrix in 1000 through bootstrap replicates.

### Statistical Analysis

Data were evaluated and statistically analyzed using Microsoft Excel (2010, version 14.0.6023.1000) software (Microsoft Corporation). A two-sample Student’s *t*-test was applied to calculate the statistical significance of differences between two sets of data. *P*-values ≤ 0.05 were considered as significant (^∗^) and *P*-values ≤ 0.005 (^∗∗^) were considered as highly significant.

## Results

### The Anti-*Candida* Activity of Synthetic PAF γ-Core Peptides

PAF contains a γ-core motif in a dextromeric isoform (GKCTKSKNEC) at its N-terminus, comprising aa 5–14 (**Figure 1A**). Thus, the γ-core is located in loop 1 that connects the first two β-strands of PAF and shows typical γ-core characteristics: a positive net charge (+1.9 at pH 7) and the participation of two Cys in two distinct disulfide bonds (Yount and Yeaman, 2004; Batta et al., 2009; **Figure 1A**).

We synthesized the 14 aa long peptide KYTGKCTKSKNECK spanning the γ-core motif of PAF and applied N-terminal acetylation and C-terminal amidation to (i) mimic the native protein backbone, (ii) to remove terminal charges, and (iii) to increase the stability against N- and C-terminal enzymatic degradation (Kim and Seong, 2001; Arnesen, 2011). Since the peptide fragment contains two Cys, we first tested to which extent the Cys in reduced or oxidized form influences the peptide function. Therefore, we synthesized three peptide variants: Pγ_red with reduced Cys having free sulfhydryl (–SH) groups, Pγ_ox with oxidized Cys forming an intramolecular disulfide bond, and Pγ_prot in which the protecting group Acm (Isidro-Llobet et al., 2009) were attached to both Cys to prevent post-synthetic oxidation of the sulfhydryl groups (**Figure 1B**). The correct peptide synthesis, Cys modification, and purity of the samples were verified by ESI-MS and RP-HPLC (Supplementary Figure S1). The antifungal activity of these three peptides was determined in broth microdilution assays on the opportunistic human pathogenic yeast *C. albicans*. All three peptides showed anti-*Candida* activity *per se*, whereby the MIC of Pγ_red was 10 μM and only twice as high as the MIC of PAF (5 μM). The MICs of Pγ_ox and Pγ_prot, however, were comparable (40 μM), but eight times higher than the PAF MIC. This indicated that Pγ_red was the most active peptide form. Based on this result we performed all further studies with the reduced peptide variant, which we termed Pγ for reasons of simplicity.

For plant derived AMPs, physicochemical properties were found to be important mediators of the antifungal activity (Sagaram et al., 2011). *In silico* analysis showed that Pγ mainly consisted of neutral and basic aa (Supplementary Table S4). Therefore, we substituted specific aa in Pγ to increase the number of basic aa, which elevated the positive net charge and reduced the GRAVY. Thereby, we created the peptide variants: Pγ^var^ (KYTGKCYKKKNECK) and Pγ^opt^ (KYTGKCKTKKNKCK) (**Figure 1C** and Supplementary Table S4). MS and RP-HPLC proved the correct synthesis and the purity of both peptides (Supplementary Figure S2). ECD spectroscopy was performed to analyze solution structural properties of Pγ, Pγ^var^, or Pγ^opt^. Expectedly, ECD spectroscopy indicated an unordered structure for all three peptides at RT, lacking any canonical secondary structural elements (Supplementary Figure S3).

The antifungal activity of these peptide variants was compared with that of Pγ and PAF on *C. albicans*. Pγ^var^ had four times higher efficacy (MIC 2.5 μM) than Pγ (MIC 10 μM), and Pγ^opt^ (MIC 1.25 μM) was even eight times more effective than Pγ. Pγ^var^ and Pγ^opt^ were two times and four times more effective than PAF, respectively (**Table 1**).

**Table 1:** MICs of PAF peptide and protein variants.

	AMP	MIC
	Pγ	10.0
Peptides	Pγ^var^	2.5
	Pγ^opt^	1.3
	PAF	5.0
Proteins	PAF^var^	1.3
	PAF^opt^	1.3

Finally, we questioned whether the aa sequence or the physicochemical features determine the activity of the peptides. Therefore, we synthesized the scrambled peptide analogs Pγ_scr (CTNKYSCKKGETKK), Pγ^var^_scr (TNGKKYKKKEYCCK), and Pγ^opt^_scr (TCKKNKKKKYGKTC). These peptides were generated by randomly shifting the aa positions in Pγ, Pγ^var^, and Pγ^opt^^3^, while keeping the overall physicochemical properties of the original peptides. The correct synthesis and the purity of the scrambled peptides were proved with MS and RP-HPLC (Supplementary Figure S4). Interestingly, all scrambled peptide variants had the same antifungal activity on *C. albicans* as Pγ, Pγ^var^, and Pγ^opt^, respectively (data not shown).

These results indicate that the primary structure plays a subordinate role, as long as the physicochemical properties (positive net charge and hydrophilicity) are maintained.

### Improvement of the Antifungal Activity of PAF by γ-Core Variation

In the next step, we exchanged the same aa in the γ-core of PAF as we did in Pγ^var^ and Pγ^opt^ (**Figure 1C**) to study the impact of the physicochemical changes of this motif on the activity and structure of the full-length protein. To achieve our objective, we generated the following PAF variants: (i) PAF^var^ by substitution of threonine to tyrosine at position 8 and serine to lysine at position 10 (T8Y S10K); and (ii) PAF^opt^, which corresponded to PAF^var^, with threonine to lysine at position 8 and the additional substitutions lysine to threonine at position 9 and glutamic acid to lysine at position 13 (T8K K9T S10K E13K). *In silico* analyses predicted the increase of the positive net charge and the hydrophilicity of the full-length protein after the specific aa exchanges (**Table 2**).

**Table 2:** Physicochemical features of PAF and its protein variants.

	PAF	PAF^var^	PAF^opt^
Substitution	None	T8Y S10K	T8K K9T S10K E13K
Charge (pH 7)	+4.7	+5.7	+7.7
GRAVY	-1.38	-1.44	-1.44

*Amino acid substitutions, the predicted net charge (at pH 7, http://protcalc.sourceforge.net) and GRAVY (www.gravy-calculator.de) are indicated.*

To generate the PAF protein variants PAF^var^ and PAF^opt^, site-directed mutagenesis was applied in the *paf* gene. The proteins were produced using the *P. chrysogenum* expression system as described earlier (Sonderegger et al., 2016). The purity of PAF^var^ and PAF^opt^ was checked by SDS–PAGE and silver staining (data not shown), and the protein identity was proved by ESI-MS (Supplementary Figure S5). The single peaks of 6,345.98 (PAF^var^) and 6,282.98 Da (PAF^opt^) confirmed the correctness of the aa exchanges and also proved the proper processing of the proteins and the formation of three intramolecular disulfide bonds in each protein variant (Supplementary Figure S5).

Antifungal susceptibility tests with *C. albicans* revealed the increased activity of both PAF variants: PAF^var^ and PAF^opt^ exhibited the same MIC (1.3 μM), which corresponded to the MIC of Pγ^opt^ (**Table 1**) and was four times lower than that of PAF (MIC 5 μM).

These results corroborated that the γ-core motif plays a central role in the antifungal activity of PAF, which can be significantly improved with the increase in positive net charge and reduction of the GRAVY. However, our data show that the increment in the positive net charge from +5.7 of PAF^var^ to +7.7 of PAF^opt^ could not further improve the anti-*Candida* efficacy of PAF^opt^. This indicates that other parts of the proteins may superimpose the physicochemical features and in consequence the function of the γ-core motif.

### Structure and Thermal Stability of PAF^var^ and PAF^opt^

**FIGURE 2:**
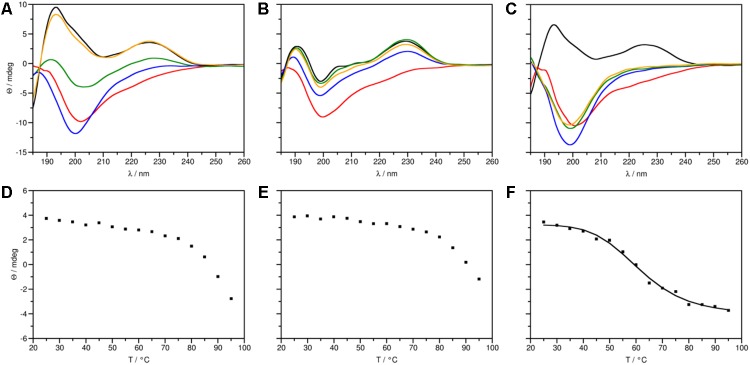
ECD spectra (top panels) and thermal unfolding curves (bottom panels) of PAF protein variants. **(A, D)** PAF [data taken from Sonderegger et al. (2016)], **(B, E)** PAF^var^ and **(C, F)** PAF^opt^. **(A–C)** Spectra in the 185–260 nm region were measured at 25 (black), 95 (red), and at 25°C directly (blue), 72 h (green), and 4 weeks (orange) after annealing. **(F)** PAF^opt^: *T*_m_ = 61.39°C, *R*^2^ = 0.9936.

ECD spectroscopic measurements were performed to compare secondary structures of the generated proteins, PAF^var^ and PAF^opt^, with the native PAF (**Figure 2A**). The ECD spectrum of PAF^opt^ recorded initially at 25°C (**Figure 2C**) was highly similar to that reported for PAF earlier (Fizil et al., 2015; Sonderegger et al., 2016), with contributions emerging from β-conformation (195, 210 nm) and disulfide bonds (228 nm). Direct comparison of spectra, however, revealed a slightly lower intensity of β-sheet contributions in the case of PAF^opt^ compared to PAF. Deconvolution of spectra using the CDSSTR method (Sreerama and Woody, 2000) indicated a 19% decrease of β-sheet content in PAF^opt^ (Supplementary Table S5). Thermal unfolding experiments followed by ECD further corroborated, that the native fold of PAF was slightly destabilized in PAF^opt^. As opposed to the melting temperature (*T*_m_) measured earlier for PAF and other members of this protein family (Sonderegger et al., 2016; Tóth et al., 2016), the *T*_m_ of PAF^opt^ was significantly lower (*T*_m_ = 61.4°C, **Figure 2F**). Furthermore, thermal unfolding of PAF^opt^ was irreversible, while slow gradual structural reorganization was observed for PAF (**Figures 2A,B**). The spectra measured for PAF^var^ indicated that these aa exchanges severely affected the secondary structure of this protein variant. The presence of disulfide bonds was apparent, but the β-strands were largely disrupted (**Figure 2B**). Deconvolution of the experimental spectrum of PAF^var^ to determine contributions emerging from canonical secondary structures was not possible. The stability of this structure is, however, comparable to that of the PAF, as thermal unfolding of PAF^var^ was incomplete even at 95°C (**Figure 2E**) and almost complete structural reorganization took place directly after the annealing (**Figure 2B**). Since thermal unfolding of PAF and PAF^var^ was incomplete in the applied temperature range, *T*_m_ could not be determined explicitly for these two proteins (**Figures 2D,E**). In summary, the native structure of PAF is retained in PAF^opt^, but the incorporation of one threonine and three lysine residues rendered the overall structure more flexible, whereas significant secondary structural changes were observed for PAF^var^ (Supplementary Figure S6).

### Protein and Peptide Variants Have Candidacidal Activity

The determination of the MIC concentration is a well-established, reliable method to elucidate the growth reducing effect of an antifungal compound. A standard MIC assay, however, does not discriminate between fungistatic or fungicidal activity. To gain deeper insight into the antifungal mechanism of the generated protein and peptide variants, we compared them first for their fungicidal potential. In liquid culture *C. albicans* was exposed to 1× MIC and 2× MIC concentrations of each protein and peptide. After certain time points aliquots were taken and appropriate dilutions were spread on agar plates to allow colony formation of viable cells. The cfu were counted and compared to the untreated control, which was set to be 100%. The assays revealed a dose- and time-dependent fungicidal effect for all compounds (Supplementary Figure S7). When applied at 1× MIC for 2 h, PAF showed no detectable killing activity, whereas PAF^var^ killed 43 ± 1% and PAF^opt^ 77 ± 2% of the *Candida* cells. The candidacidal effects were aggravated with higher protein concentrations. Exposure of *C. albicans* for 2 h to 2× MIC of PAF, PAF^var^, and PAF^opt^ killed 26 ± 6, 54 ± 5, and 96 ± 1% of the cells, respectively. The most potent candidacidal protein was PAF^opt^ that killed 60 ± 10% of the cells within 10 min of exposure.

The peptides Pγ and Pγ^var^ had no killing effects on *C. albicans* when applied at 1× MIC within 2 h, whereas 1× MIC of Pγ^opt^ destroyed 29 ± 10% of the cells. However, the killing efficacy increased with the application of 2× MIC of Pγ, Pγ^var^, and Pγ^opt^ to 31 ± 23, 50 ± 28, and 88 ± 8% after 2 h (Supplementary Figure S7).

**FIGURE 3:**
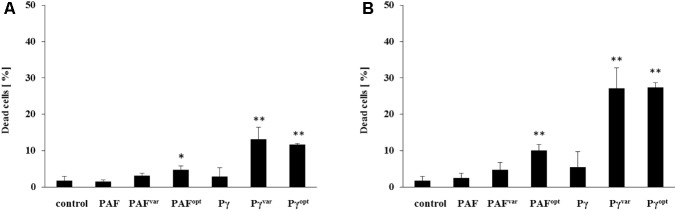
Early-stage candidacidal activities of AMPs. *C. albicans* was exposed for 10 min to **(A)** 1× MIC and **(B)** 2× MIC of AMPs and analyzed with dead-cell staining by FACS. Values represent mean ± SD; ^∗^*P* ≤ 0.05 and ^∗∗^*P* ≤ 0.005.

Although the plating method is a simple and common tool to screen for fungicidal activities, it shows certain limitations. Depending on their mode of action, AMPs may still cause cell death beyond the exposure time and during plate-incubation, e.g., when AMPs are taken up and act inside the fungal cell, as it was shown to be the case with PAF (Oberparleiter et al., 2003; Sonderegger et al., 2017; Huber et al., 2018). To elucidate the AMPs candidacidal activities at an early stage and to compare their time and dose-dependent cell killing in more detail, fluorescence activated cell sorting (FACS) analysis with dead-cell staining was applied. To this end, *C. albicans* was exposed to 1× and 2× MIC of the respective AMPs for 10 min, stained with the dead-cell marker, and fixed with formaldehyde. With FACS analyses, 1.8 ± 1.2% dead cells were detected in the untreated control (**Figure 3**). PAF and PAF^var^ did not cause significant increase in this number after 10 min of exposure, neither when applied at 1× MIC nor at 2× MIC. PAF^opt^ killed 4.8 ± 1% (*P* = 0.027) of the cells at 1× MIC and 10.1 ± 1.7% (*P* = 0.002) at 2× MIC within the same time. No significant cell-killing could be observed with the peptide Pγ at both concentrations tested. In contrast, the peptides Pγ^var^ and Pγ^opt^ showed very similar and improved efficacy: at 1× MIC, Pγ^var^ and Pγ^opt^ killed 13.2 ± 3.2% (*P* = 0.005) and 11.7 ± 0.3% (*P* < 0.001) of the cells, respectively. When applied at 2× MIC Pγ^var^ killed 27.1 ± 5.6% (*P* = 0.002) and Pγ^opt^ killed 27.4 ± 1.3% (*P* < 0.001) of the *C. albicans* cells (**Figure 3**).

**FIGURE 4:**
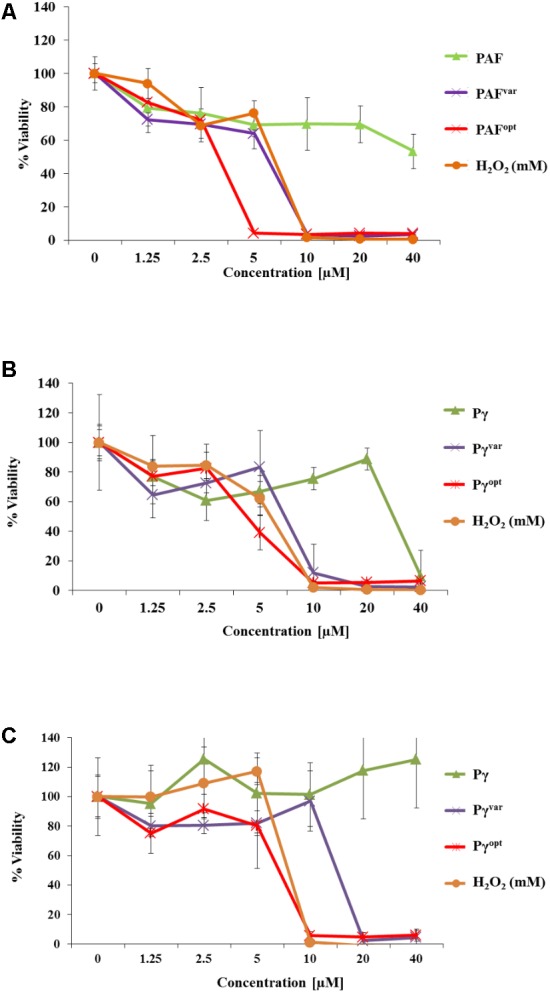
Anti-biofilm activities of AMPs. In 96-well microtiter plates *C. albicans* was allowed to establish a biofilm for **(A,B)** 1 h and **(C)** 24 h before 0–40 μM of **(A)** protein and **(B,C)** peptide variants were added. H_2_O_2_ (0–40 mM) served as a positive control. Values represent the mean of three replicates. Values represent mean ± SD.

Next, we analyzed the potential of the AMPs to inhibit *C. albicans* biofilm formation. Low susceptibility of biofilms to antifungal agents is often a limiting factor in anti-*Candida* treatment. To this end, *C. albicans* cells were distributed in 96-well microtiter plates, allowed to settle down, and to develop biofilms for 1 and 24 h. Then, planktonic cells were washed off and AMPs were added to the biofilms for another 24 h in a concentration range of 0–40 μM. The biofilms were then quantified by adding FDA to the wells. Metabolically active cells convert non-fluorescent FDA into highly fluorescent fluorescein by intracellular enzymatic activity (Peeters et al., 2008). The resulting fluorescence signal intensity from AMP-treated biofilms was measured and compared to untreated conditions. The extinction of FDA fluorescence in 1 h old biofilms exposed to 10 μM PAF^var^ and 5 μM PAF^opt^ indicated that both proteins exhibited potent anti-biofilm activity (**Figure 4**). In contrast, PAF only moderately reduced the metabolic activity of young biofilms when applied in concentrations up to 40 μM (**Figure 4**). Twenty-four-hour-old biofilms, however, were resistant against PAF, PAF^var^, and PAF^opt^ in the investigated concentration range (data not shown).

The peptide Pγ exhibited activity on young biofilms at 40 μM, but had no impact on 24-h-old biofilms (**Figure 4**). Pγ^var^ and Pγ^opt^ showed inhibitory activity on young biofilms at a concentration of 10 μM, respectively, and inhibited cell metabolism even in 24-h-old biofilms at 10 μM (Pγ^opt^) and 20 μM (Pγ^var^) (**Figure 4**).

### Candidacidal Activity Associates With Cell Shrinkage and ROS Formation

**FIGURE 5:**
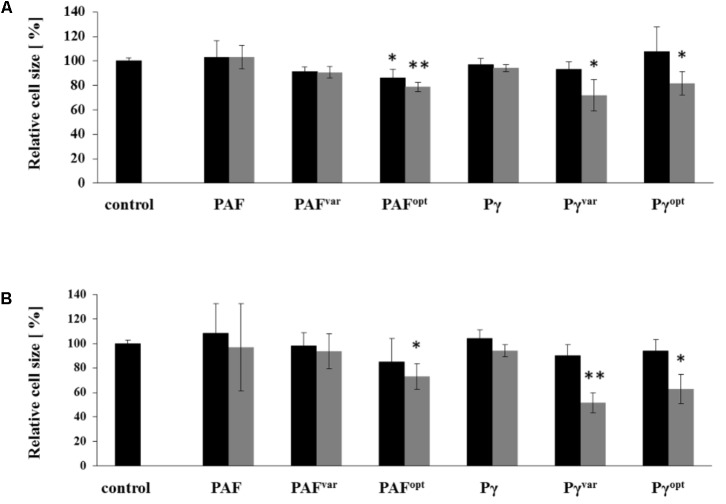
Cell size reduction by AMPs. *C. albicans* cells were treated for **(A)** 10 and **(B)** 30 min with 1× MIC (black bars) and 2× MIC (gray bars) and analyzed by flow cytometry. The diagram shows the change in FSC values compared to untreated control cells. Values represent mean ± SD; ^∗^*P* ≤ 0.05 and ^∗∗^*P* ≤ 0.005.

Cell shrinkage of *C. albicans* is a sign for cell death when exposed to AMPs (Lee et al., 2017). We therefore analyzed the changes in size of AMP-treated *C. albicans* cells by recording the FSC with flow cytometry. To this end, *C. albicans* cells were analyzed after 10 and 30 min of exposure to 1× MIC and 2× MIC of AMPs (**Figure 5**). Heat-killed and untreated *C. albicans* cells served as positive and negative controls, respectively (Supplementary Figure S8). The cell size of untreated cells slightly increased during incubation time, which pointed at proliferation by cell budding (Supplementary Figure S8). At 1× MIC the proteins PAF^var^ and PAF^opt^ caused only a minor reduction in cell size, but when applied at 2× MIC cells size reduction was further increased with PAF^opt^ by 21 ± 4% (*P* = 0.002) and 27 ± 10% (*P* = 0.011) after 10 and 30 min, respectively. Exposure to PAF and Pγ did not result in cell shrinkage, confirming the fungistatic character of these AMPs (compare **Figure 3**). Pγ^var^ and Pγ^opt^ caused a very prominent decrease in the FSC value compared to the untreated cell population when applied at 2× MIC. Pγ^var^ and Pγ^opt^ reduced the average cell size after a 10-min treatment by 28 ± 13 (*P* = 0.022) and 18 ± 10% (*P* = 0.041), respectively, and after 30 min the cell size was reduced by 48 ± 8 (*P* < 0.001) and 37 ± 12% (*P* = 0.007), respectively.

In previous studies, we could show that the activity of PAF in filamentous fungi is closely linked to the formation of ROS (Kaiserer et al., 2003; Marx et al., 2008). Since ROS production is an early marker for programmed cell death, we were also interested whether the investigated AMPs induce ROS in *C. albicans*. Therefore, *C. albicans* cells were loaded with DCFH-DA, which is converted intracellularly to the highly fluorescent DCF when oxidized in the presence of ROS (Chen et al., 2010). During exposure of the cells to 0–20 μM AMPs the DCF signal intensities were recorded in RFU every 5 min over a time-course of 4 h. The background RFU of 6,757 ± 117 was detected in *C. albicans* cells that were not exposed to AMPs, but loaded with DCFH-DA, diluted in 0.05× PDB (0.1% DMSO final concentration). The RFU value remained constant over the time of measurement. For all AMPs, we detected a time- and dose-dependent increase of ROS (Supplementary Figure S9) and ROS production was prevented by the addition of 1 mM of the antioxidant ascorbic acid to the highest concentration (20 μM) of each AMP (Supplementary Figure S10).

**FIGURE 6:**
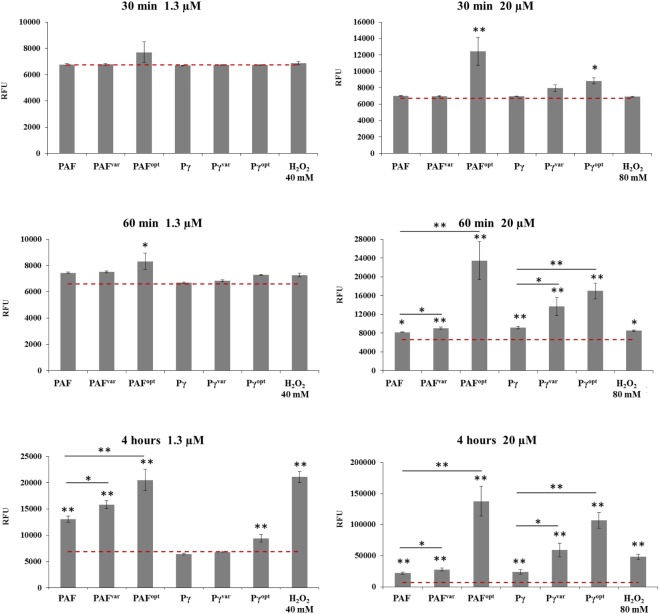
ROS production in *C. albicans* after 0.5, 1, and 4 h of AMP treatment. Cells were exposed to 1.3 and 20 μM AMP. The dotted red line indicates the background RFU signal of untreated cells (negative control). H_2_O_2_ was used at 40 and 80 mM as positive control. Values represent mean ± SD; ^∗^*P* ≤ 0.05 and ^∗∗^*P* ≤ 0.005.

Significant differences in ROS induction between the AMPs were visible when applied at high concentrations and/or with long incubation times. After 30 min of exposure to 1.3 μM AMPs no significant ROS induction was apparent (**Figure 6**). After 60 min, 1.3 μM PAF^opt^ induced a fluorescence signal of 26% (*P* = 0.04) above the background. After 4 h of incubation, significant ROS production was triggered by PAF, PAF^var^, and PAF^opt^ at 1.3 μM. PAF induced a DCF signal of 13,057 ± 612 (*P* < 0.001) RFU (**Figure 6**). The protein variants induced even more oxidative stress than PAF: PAF^var^ and PAF^opt^ signals were 21% (*P* = 0.008) and 57% (*P* = 0.004) higher than the PAF signal, respectively. However, when the proteins were applied at 20 μM, PAF^opt^ induced a fluorescence signal of 87% (*P* = 0.006) above the background signal after only 30 min and a signal of even 255% (*P* = 0.002) above the background after 60 min. Also, PAF induced a signal at 20 μM after 1 h [8,151 ± 94 (*P* = 0.027) RFU]. However, significant higher signals were triggered with PAF^var^ (+11%, *P* = 0.005) and PAF^opt^ (+188%, *P* = 0.003), when compared to PAF. When applied for 4 h, this increase in ROS-specific signals could be even further elevated. While PAF^var^ increased the intensity signals by 26% (*P* = 0.025) above those evoked by PAF [22,116 ± 94 (*P* < 0.001) RFU], PAF^opt^ dramatically increased ROS generation by 523% (*P* = 0.001) (**Figure 6**).

Similar results were observed for the peptide variants. When applied at 1.3 μM, no significant ROS induction could be detected after 30 and 60 min. After 4 h 1.3 μM Pγ^opt^ increased ROS signals significantly 36% (*P* = 0.005) above the background. When peptides were applied at 20 μM Pγ^opt^ induced a significant signal of 31% (*P* = 0.015) above the background signal after 30 min. After 60 min, all peptides significantly increased the ROS levels. Whereby, compared to Pγ [9,158 ± 293 (*P* = 0.006) RFU], the signals caused by Pγ^var^ (+50%, *P* = 0.016) and Pγ^opt^ (+85%, *P* = 0.001) were significantly higher. When 20 μM was applied for 4 h, Pγ^var^ raised the ROS signals to 146% (*P* = 0.007) and Pγ^opt^ to 446% (*P* < 0.001) above the level induced by Pγ [24,025 ± 4072 (*P* = 0.002) RFU] (**Figure 6**).

Based on these observations we conclude that the AMPs are potent ROS-inducing molecules. The highest ROS levels were observed after 4 h in *C. albicans* treated with PAF^opt^ and the synthetic peptide Pγ^opt^ (**Figure 6**), which showed to be the AMPs with the highest fungicidal activity (**Figure 3** and Supplementary Figure S7).

This finding corroborates that the mode of action of the *P. chrysogenum* AMPs is closely linked with ROS production not only in filamentous fungi but also in yeast cells. Importantly, the γ-core motif *per se* triggers ROS production. The level of ROS generation, however, depends on the physicochemical features of this structural motif in the proteins and peptides.

### Hemolysis and Cytotoxicity

Whenever a therapeutic application of a compound is considered it is crucial to rule out any detrimental side effects in the host such as hemolysis or cytotoxicity. Therefore, we first tested the AMPs for hemolytic activity against erythrocytes on blood agar. None of the investigated AMPs caused hemolysis at the concentration tested (20 μg per filter disc) (Supplementary Figure S11).

To further elucidate the cytotoxic potential, we performed *in vitro* viability staining of the human skin cells with PI after exposure to 30 μM of AMPs for 16 h. The nucleus-specific fluorescent stain Hoechst 33342 was used as counterstain. Fluorescence microscopy revealed no increase in PI-positive cells after AMP treatment at any condition tested (Supplementary Figure S12).

### The γ-Core Motif Is a Phylogenetic Trait of Small, Cysteine-Rich, Cationic Proteins in Eurotiomycetes

**FIGURE 7:**
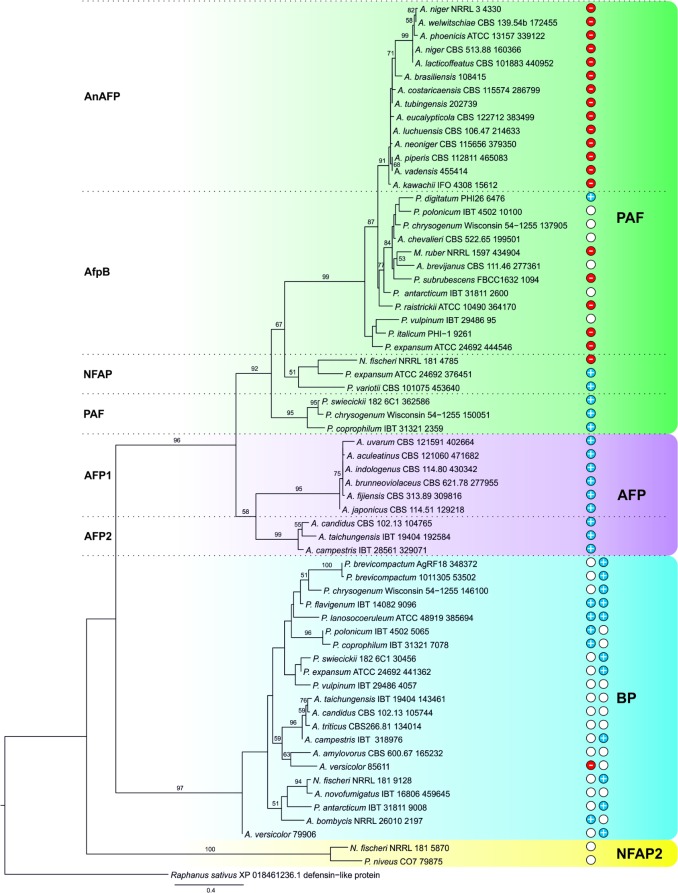
Maximum-likelihood (ML) tree of putative AMPs from Eurotiomycetes. ML bootstrap values >50% are shown next to branches. The isolate names are given with the accession number of the respective AMP (listed in Supplementary Table S6). The net charge(s) (at pH 7.0) of the γ-core motif(s) are indicated: Blue discs with “+” indicate positive (≥ +0.8), red discs with “-” negative (≤ –0.9), and white discs the almost neutral (–0.2 to +0.3) γ-cores. After BP proteins the net charge of middle and the C-terminal γ-core is shown from left to right. Proteins belonging to PAF-, AFP, BP-, and NFAP2-clades are in green, lilac, blue, and yellow background, respectively.

**FIGURE 8:**
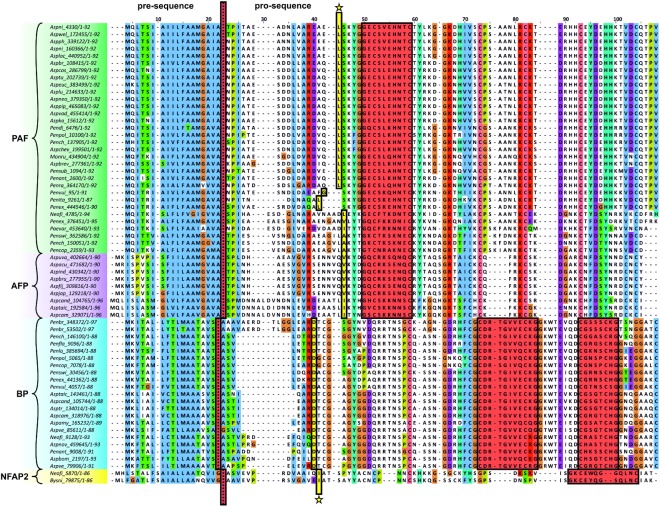
Clustal W multiple alignment of the putative AMPs from Eurotiomycetes. Dotted red line indicates the cleavage site of the predicted signal sequence. First amino acid of the mature protein is marked by an asterisk. The γ-core motifs are framed and highlighted in red. Abbreviation of the species name and the accession number of the respective AMP (listed in Supplementary Table S6) are indicated. The Clustal X default color scheme was applied (http://www.jalview.org/help/html/colourSchemes/clustal.html).

Our study corroborates a major impact of the PAF γ-core on the antimicrobial action and suggests an evolutionary importance. We propose that AMPs are commonly present in the genome of Eurotiomycetes, and the γ-core motif is not restricted to the PAF-related proteins, but can be found in phylogenetically distinct, cysteine-rich AMPs (Paege et al., 2016). The MycoCosm portal (Grigoriev et al., 2014) contains 167 genomes of Eurotiomycetes representing 153 species. BLAST searches in this portal yielded 40 *P. chrysogenum* PAF-, 10 *A. giganteus* antifungal protein AFP-, 21 *P. brevicompactum* “BP”-, and only 2 *N. fischeri* NFAP2-related proteins. These are separated into four major clades with strong statistical support (bootstrap values ≥ 90) in the constructed phylogenetic tree (**Figure 7**). Within the PAF-clade, the proteins homolog to *A. niger* antifungal protein (AnAFP), *P. digitatum* AfpB, *N. fischeri* NFAP, and PAF form separated subclades (**Figure 7**). Similarly, two subclades of AFP-related proteins can be distinguished (**Figure 7**). The presence of AMPs is mainly restricted to aspergilli and penicilli, but a *Monascus* strain has a PAF and a *Paecilomyces* strain a PAF and a NFAP homolog (Supplementary Table S6). We did not find any protein with significant similarity to the applied template AMPs in the genome of *Arthroderma*, *Blastomyces*, *Byssochlamys*, *Caliciopsis*, *Capronia*, *Cladophialophora*, *Coccidioides*, *Coccodinium*, *Cyphellophora*, *Endocarpon*, *Eurotium*, *Exophiala*, *Fonsecaea*, *Gymnascella*, *Histoplasma*, *Microsporum*, *Paracoccidioides*, *Phaeomoniella*, *Phialophora*, *Talaromyces*, *Thermoascus*, *Thermomyces*, *Trichophyton*, and *Uncinocarpus* species. The position of the γ-core motif is conserved within the major clades. However, their aa composition varies. A dextromeric γ-core isoform is localized in PAF- and AFP-related proteins at the N-terminus, in NFAP2-related proteins at the C-terminus (**Figure 8**). Interestingly, BP homologs contain two levomeric γ-core motifs: a short one at the C-terminus and a long one in the middle of the protein (**Figure 8**). From the analysis of the physicochemical properties of the γ-core motifs, we conclude that all of them are hydrophilic, except for the short C-terminal hydrophobic γ-core motif of BP from *Aspergillus amylovorus* (Supplementary Table S6). AMPs from Eurotiomycetes show γ-core motifs with positive, negative, or almost neutral net charge. The proteins of the AnAFP subclade exhibit negatively charged γ-cores, those of the AFP clade and PAF subclade contain positively charged γ-cores, and those of the NFAP2 clade neutral γ-cores (**Figure 8** and Supplementary Table S6).

## Discussion

The conservation of the γ-core motif in proteins with antimicrobial activity over an evolutionary period of 2.6 billion years implicates a central functional role (Yount and Yeaman, 2006). The role of the γ-core motif of AMPs from ascomycetes is not understood, although this motif was assigned a common structural feature of the members of PAF- and AFP-clades before (Paege et al., 2016). Our very detailed screening and statistically well-supported phylogenetic analysis (bootstrap values ≥ 90 in **Figure 7**) revealed that the γ-core motif is not restricted to PAF and AFP homologs, but can also be found in BP- and NFAP2-related proteins (**Figure 8**). We showed for the first time that they are localized at conserved positions within a major clade suggesting its structural and/or functional importance. This hypothesis is strengthened by the observation that the γ-core determines the antifungal activity of the plant defensins MsDef1 and MtDef4 depending on the positive net charge (Sagaram et al., 2011). However, it has to be noted that the γ-core of Eurotiomycetes AMPs can be positively, negatively charged, or have an almost neutral charge (–0.2 – +0.3 at pH 7), which may influence their antifungal activity and their mode of action.

So far, no reports exist on antifungal active γ-core peptides from ascomycetes AMPs. This study is therefore the first on the synthetic peptide Pγ spanning the γ-core motif of PAF that shows significant activity against *C. albicans* at a MIC only twofold higher than the MIC of the full-length protein. This underlines the important role of the γ-core motif for the antifungal function of PAF, but additionally demonstrates that other protein motifs may contribute to full antifungal activity.

In PAF and in the closely related AfpB the positively charged γ-core is present in a dextromeric isoform at the N-terminus of the proteins. When the γ-core of AfpB was investigated the synthetic γ-core peptide was cyclized by stabilizing its structure with an intramolecular disulfide bond (Garrigues et al., 2017). Considering the observed antifungal inactivity of macrocyclic AfpB γ-core peptide and the observation that structural flexibility is an important activity determinant for antimicrobial peptides (Liu et al., 2013), we paid special attention to these aspects in the present study. We synthesized the PAF γ-core peptides with the Cys in three different chemical configurations and proved that Pγ_red containing reduced Cys exhibited the highest antifungal activity, whereas the peptides with oxidized or protected Cys (Pγ_ox and Pγ_prot, respectively) proved to be less active against *C. albicans*. The γ-core motif contributes to the formation of loop 1 in PAF allowing flexibility to some extent (Batta et al., 2009). Furthermore, the two Cys within the γ-core motif are not connected with each other but they participate in disulfide bonds with Cys of the neighboring β-strands (Fizil et al., 2015). The intramolecular disulfide bond in Pγ_ox resulted in a macrocyclic peptide structure with limited conformational flexibility compared to the parent linear peptide Pγ. The flexibility of Pγ_prot should correspond to that of Pγ_red due to the small size of the Acm protecting group (Isidro-Llobet et al., 2009). Nevertheless, its antifungal activity was less, which cannot be explained with reduced structural flexibility. Therefore, we hypothesize that the added acetamido groups disturbed the biological function of the peptide by impeding molecular interactions.

By varying the aa composition of the peptide Pγ to increase the positive net charge and hydrophilicity the antifungal activity of Pγ^var^ and Pγ^opt^ could be significantly enhanced. Therefore, it seems plausible that the NFAP2-derived γ-core peptide did not possess antifungal activity since it was slightly negatively charged (-0.2) and more hydrophobic (GRAVY = -0.373) than the positively charged and highly hydrophilic Pγ (Tóth et al., 2018). The AfpB-derived γ-core peptide was positively charged (+1.1) with a GRAVY of -0.718 (Garrigues et al., 2017), though less charged and hydrophilic than Pγ. This could be a further explanation for its lack of antifungal activity in addition to its macrocyclic structure (Garrigues et al., 2017). Another well-studied example is the synthetic antifungal peptide PAF26. When the positive net charge was partially or fully neutralized by aa substitution, the antifungal activity of PAF26 decreased (Muñoz et al., 2007, 2013). It can be assumed that the physicochemical properties (such as positive net charge and hydrophilicity) together with the high structural flexibility regulate the attraction of γ-core peptides to the fungal cell membrane, which is more negatively charged than a human cell membrane. The attraction to the membrane does not *per se* imply a membrane targeted and/or membrane disruptive activity of the peptide (Wilmes et al., 2011), but may be an important first step that determines a more distinct mode of action (Kaiserer et al., 2003; Oberparleiter et al., 2003; Hegedüs et al., 2011; Sonderegger et al., 2017; Huber et al., 2018). When interacting with a potential target molecule the primary structure may be negligible for this kind of short, unstructured, and flexible peptides, as we could prove that the scrambled peptide variants showed the same antifungal activity as the parental peptides. This was found to be true also for NFAP2-derived synthetic peptides (Tóth et al., 2018).

Nowadays, the synthesis of small peptides resistant to the proteolytic degradation can be economic (Ciociola et al., 2016); nevertheless, the generation of highly active and stable full-length PAF variants provides certain advantages over short synthetic antifungal peptides. In the present study, we underlined the applicability of the previously developed *P. chrysogenum* based-expression system (Sonderegger et al., 2016) in the production of the full-length PAF γ-core variants PAF^var^ and PAF^opt^. The elevation of the hydrophilicity of PAF by altering the γ-core aa sequence similarly improved the antifungal activity of the protein as for the respective peptides. Interestingly, PAF^var^ and PAF^opt^ have the same GRAVY and inhibited the growth of *C. albicans* at the same MIC, irrespectively of different net charges (PAF^var^: +5.7; PAF^opt^: +7.7). This indicates that an increase in positive net charge to +7.7 did not further improve the antifungal activity of the protein in contrast to observation with the peptides Pγ^var^ and Pγ^opt^. The overall surface charge distribution of PAF is important for full antifungal activity. Distinct changes in the surface charge by aa exchanges are even relayed to distant protein regions (Sonderegger et al., 2017). Therefore, it is reasonable to assume that other parts/motifs of the protein superimpose the net charge of the γ-core in PAF. Interestingly, the ECD measurement of PAF^opt^ revealed an altered and more flexible protein structure compared to PAF. Based on the observations that structural flexibility of distinct protein motifs can enhance the specific protein activity (Wallnoefer et al., 2010), we cannot exclude that the increased flexibility of PAF^opt^ enhances antifungal activity, but this is attenuated by other protein parts. It may be worth to perform detailed NMR-based structural analyses of this protein variant, which may expand our knowledge about the mechanistic function of PAF.

By investigating the mode of action of the AMPs we could show that the improved protein and peptide variants act fungicidal on *C. albicans*, which is associated with cell shrinkage and ROS formation. In the filamentous fungus *A. nidulans* ROS-associated apoptosis induction by PAF was shown before (Leiter et al., 2005). In the present study, we observed for the first time that PAF and the derived AMPs elicit ROS in the yeast *C. albicans*, which points toward a similar mode of action on both fungal species. Notably, ROS (e.g., H_2_O_2_) has been associated with the induction of *C. albicans* filamentation, which represents a virulence attribute (Srinivasa et al., 2012; Su et al., 2018). None of the investigated AMPs triggered the formation of pseudohyphae or hyphae in *C. albicans* under the applied test conditions (Supplementary Figure S13).

Since ROS formation and cell shrinkage are also associated with apoptosis in yeast (Perrone et al., 2008; Munoz et al., 2012), we propose that PAF and the derived AMPs induce apoptosis in *C. albicans*. However, further experiments will be needed to confirm this assumption. It also has to be noted that the fluorescence probe DCFH-DA does not exclusively detect ROS, but can also be converted into DCF by reactive nitrogen species (RNS). It cannot be distinguished to which extend DCF is formed by ROS or RNS (Chen et al., 2010).

A rapid and increased fungicidal activity, which was the highest for Pγ^opt^ and PAF^opt^, is particularly important to counteract resistance development in target organisms (Duncan and O’Neil, 2013). For example, the formation of multi-drug resistance was recently observed in *C. albicans*, where a clinically isolated strain showed combined resistance against fluconazole, caspofungin, and amphotericin B (Sanglard, 2016). These three drugs represent the three major drug classes of currently applied antifungal therapeutics, namely azoles, echinocandins, and polyenes (Duncan and O’Neil, 2013). Besides the occurring drug resistance in *C. albicans* the biofilm forming ability has great medical relevance. Biofilm formation of *C. albicans* on medical devices like catheters, dentures, contact lenses, prosthetic joints, and cochlear implants poses a major problem as it can be the source for severe clinical infections (Giles et al., 2018). Furthermore, biofilm formation in the host, e.g., when colonizing the skin and mucosa, hamper effective drug delivery and might favor the development of drug resistance. We could show that the PAF-related antifungal proteins and peptides investigated in this study are able to impede early-stage biofilm formation, whereby the two peptides Pγ^var^ and Pγ^opt^ showed potent inhibitory activity against mature (24 h old) biofilm. The decrease of drug efficacy during biofilm maturation was also observed for other peptide antifungals, which are trapped and/or inactivated by the extracellular matrix of mature biofilms (Duncan and O’Neil, 2013). The peptides Pγ^var^ and Pγ^opt^ are therefore interesting candidates for further anti-biofilm investigations.

In general the treatment of skin or mucosa is easier to achieve than systemic treatment (Duncan and O’Neil, 2013), because systemic administration of peptides can be hampered by low bioavailability, poor serum stability, and the risk to cause immunological reactions (Yount and Yeaman, 2012). Another limiting factor is the potential of antimicrobial peptides to be cytotoxic toward mammalian cells (Kang et al., 2017). For example, the cationic character and the α-helical structure determine the membrane lytic activity of the 26 aa long peptide melittin, a major component of the bee venom. Attempts to disturb the α-helix conformation by aa substitutions resulted in a decreased cytotoxicity, but retention of minimum antimicrobial inhibitory concentrations (Jamasbi et al., 2016). The lack of a secondary structure of the synthetic γ-core peptides investigated in our study could explain their non-toxic and non-lytic character on mammalian cells *in vitro*. However, the potential of host damage of the PAF-derived AMPs has to be investigated further by *in vivo* test in the future.

In summary, we could show that the evolutionary conserved γ-core motif of PAF plays an important role for the fungicidal activity of this small, cysteine-rich, and cationic protein and that its efficacy can be significantly improved by changing the physicochemical properties of this central motif by aa substitution based on rational design. PAF-derived proteins and peptides promise high potential against *C. albicans* biofilm formation and might pave the way toward the development of novel compounds preferentially for topical application to treat skin and/or mucosal fungal infections.

## Ethics Statement

The study with human keratinocytes and fibroblasts was carried out in accordance with the recommendations of the Ethics Committee of the Medical University of Innsbruck. The protocol was approved by the Ethics Committee of the Medical University of Innsbruck. All subjects gave written informed consent in accordance with the Declaration of Helsinki.

## Author Contributions

FM, CS, SD, GT, and DW designed the experiments, conceived, and supervised the study. GV and LG helped to design synthetic peptides, and GV synthesized and analyzed the peptides. CS prepared expression vectors and generated the recombinant proteins, analyzed the anti-*Candida* mode of action of proteins and peptides, performed cytotoxicity tests with proteins and peptides, and analyzed the data. LG and SK performed *in silico* phylogenetic analyses. CS and WP performed FACS-based experiments and analyzed the data. AB performed ECD spectroscopy and analyzed the data. All authors contributed to manuscript writing and to manuscript revision and approved the submitted version.

## Conflict of Interest Statement

The authors declare that the research was conducted in the absence of any commercial or financial relationships that could be construed as a potential conflict of interest. The reviewer JM–Á and handling editor declared their shared affiliation at time of review.

## Footnotes

1 http://www.gravy-calculator.de/

2 http://protcalc.sourceforge.net/

3 http://www.bioinformatics.org/sms2/shuffle_protein.html

## Abbreviations

Acm: acetamidomethyl
AMPs: antimicrobial peptides/proteins
DCF: 2′,7′-dichlorofluorescein
DCFH-DA: 2′,7′-dichlorodihydrofluorescein diacetate
ECD: electronic circular dichroism
ESI-MS: electrospray ionization mass spectrometry
FACS: fluorescence-assisted cell sorting
FDA: fluorescein diacetate
Fmoc: fluorenylmethyloxycarbonyl
FSC: forward scatter
GRAVY: grand average of hydropathy
HDF: primary human dermal fibroblasts
HKC: primary human keratinocytes
MIC: minimal inhibitory concentration
PAF: *Penicillium chrysogenum* antifungal protein
PI: propidium iodide
RFU: relative fluorescence units
ROS: reactive oxygen species
RP-HPLC: reversed phase high-performance liquid chromatography
SSC: side scatter
