# Supplementary material for: The Evolutionary Conserved γ-Core Motif Influences the Anti-Candida Activity of the Penicillium chrysogenum Antifungal Protein PAF
Source: Front Microbiol. 2018 Jul 20;9:1655. doi: 10.3389/fmicb.2018.01655 (PMC6062912; doi:10.3389/fmicb.2018.01655)
Supplement: Supplementary file 1 [file Data_Sheet_1.docx]

# *Supplementary Material*

**The evolutionary conserved γ-core motif influences the**

**anti-*Candida* activity of the *Penicillium chrysogenum***

**antifungal protein PAF**

**Christoph Sonderegger, Györgyi Váradi, László Galgóczy, Sándor Kocsubé, Wilfried Posch, Attila Borics, Sandrine Dubrac, Gábor K. Tóth, Doris Wilflingseder and Florentine Marx^*^**

***Correspondence:**

**Florentine Marx, PhD**

florentine.marx@i-med.ac.at


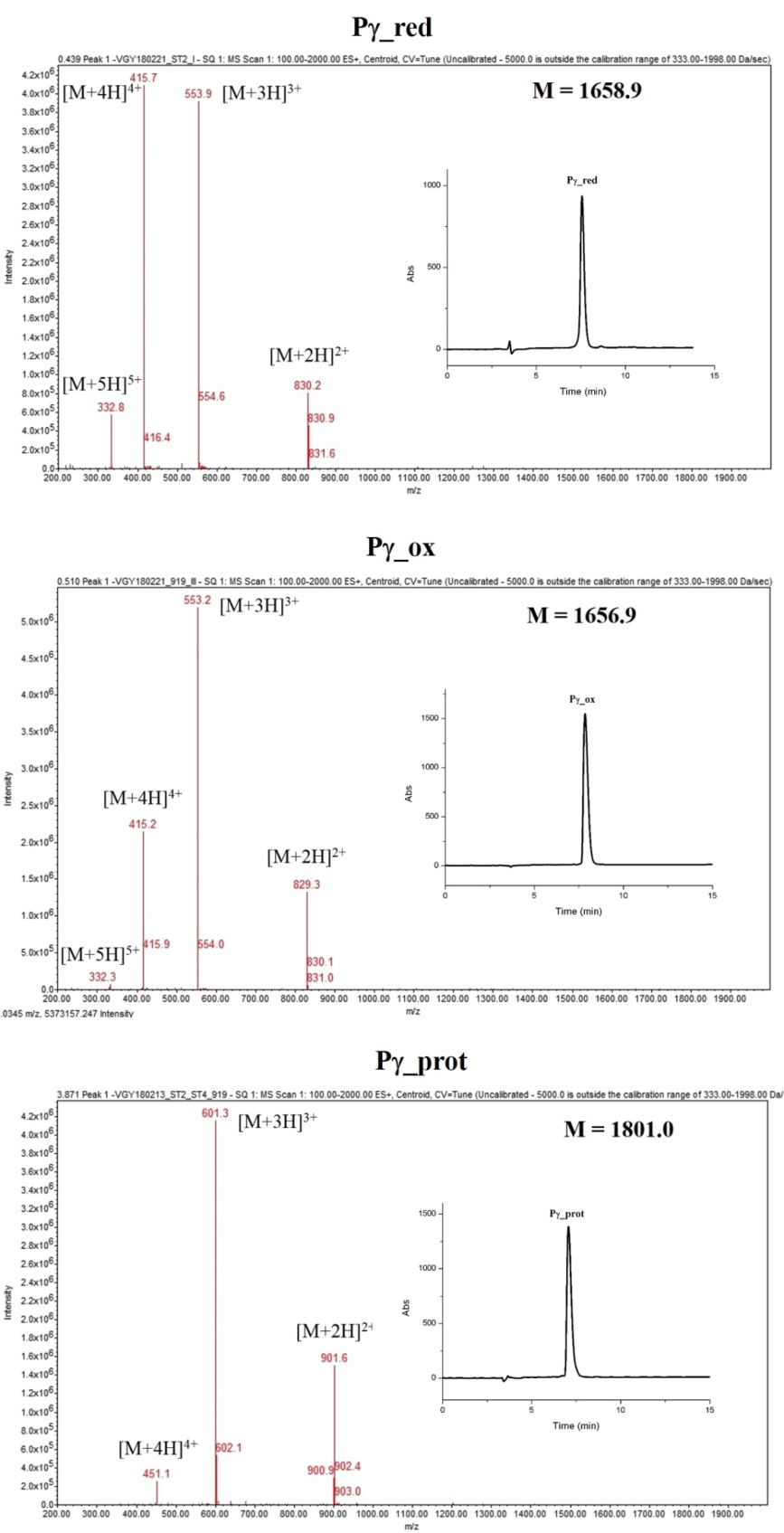


**Figure S1. Mass spectrometry and RP-HPLC data (insets) for Pγ peptides**. Shown are the three modifications of Pγ: in its reduced (Pγ_red) and oxidized form (Pγ_ox) and with protecting groups at the cysteines (Pγ_prot). Linear gradient from 12 to 27% (Pγ_red and Pγ_prot) and from 10 to 25% (Pγ_ox) solvent (B) over 15 min. Flow rate: 1.0 mL min^−1^.


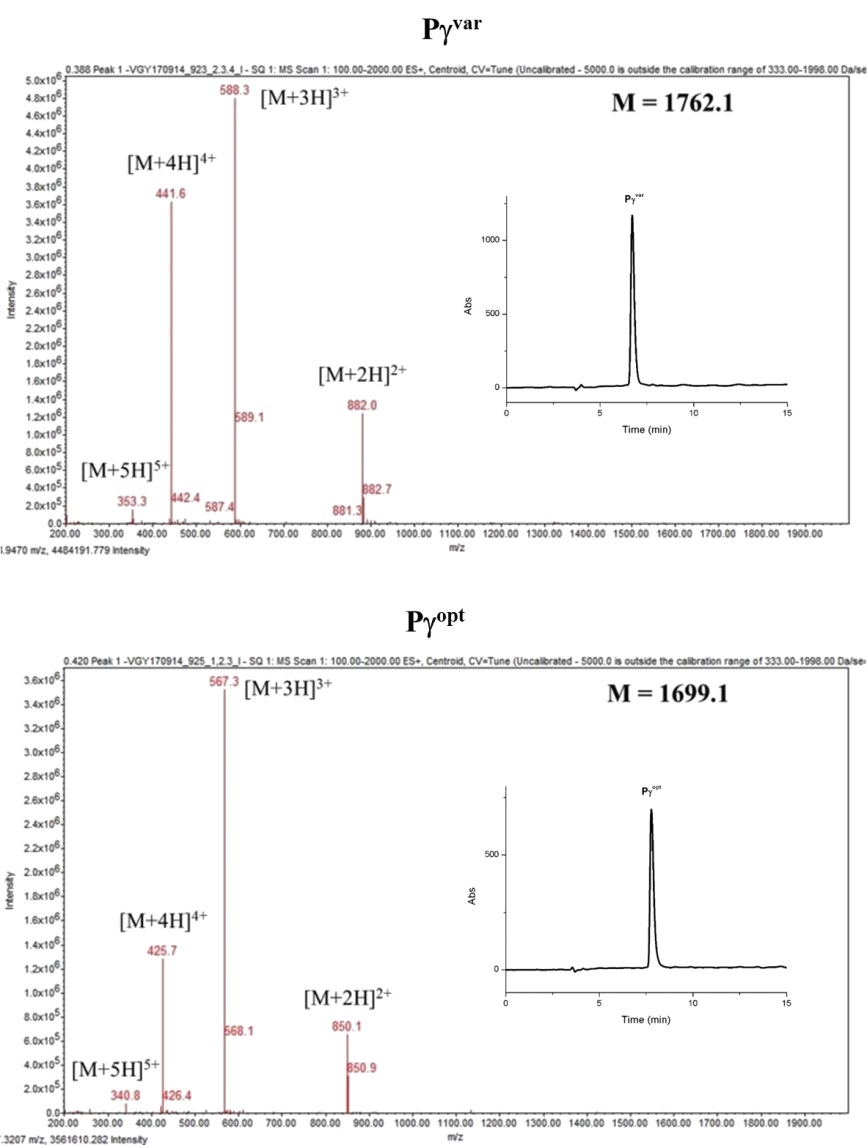


**Figure S2. Mass spectrometry data and RP-HPLC profiles (insets) for the peptides Pγ^var^ and Pγ^opt^**. Linear gradient from 14–29% (Pγ^var^) and from 11–26% (Pγ^opt^) solvent (B) over 15 min. Flow rate: 1.0 mL min^−1^.

**
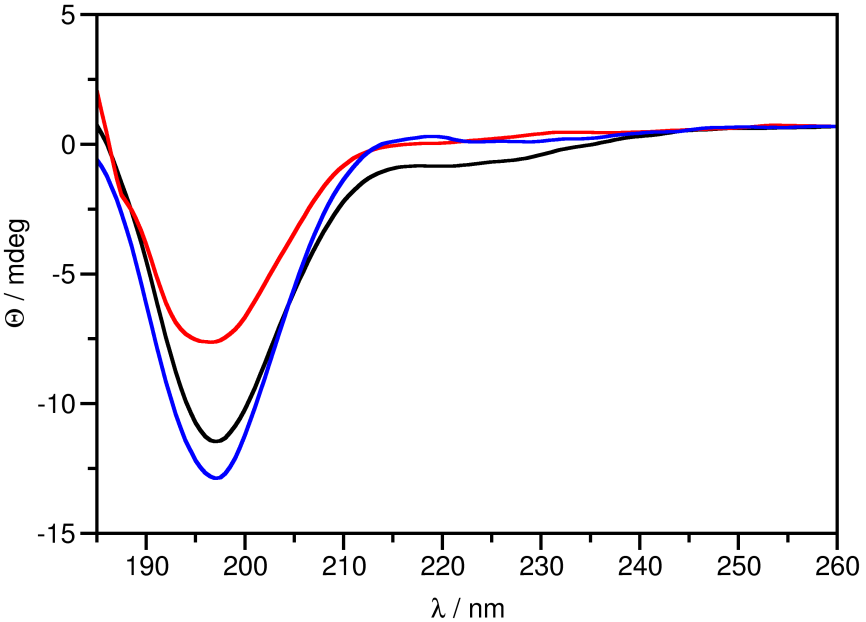
**

**Figure S3. ECD spectra of γ-core peptides.** ECD spectra of Pγ (black), Pγ^var^ (red) and Pγ^opt^ (blue) γ-core peptides, acquired in pure H_2_O at 25 ºC.


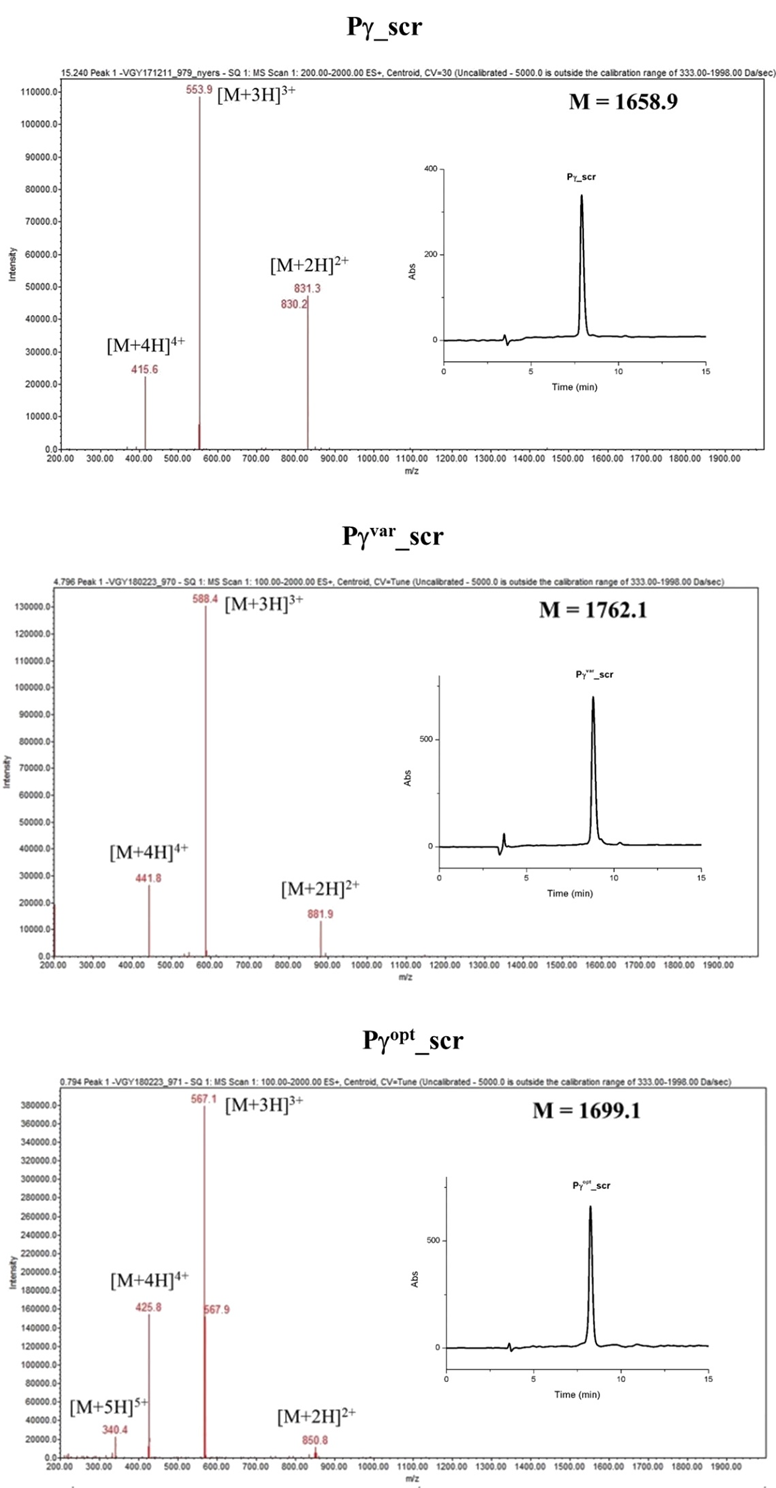


**Figure S4.** **Mass spectrometry and RP-HPLC profiles of Pγ^var^_scr and Pγ^opt^_scr.** Linear gradient from 12–27% (Pγ_scr and Pγ^var^_scr) and from 10–25% (Pγ^opt^_scr) solvent B over 15 min. Flow rate: 1.0 mL min^−1^.


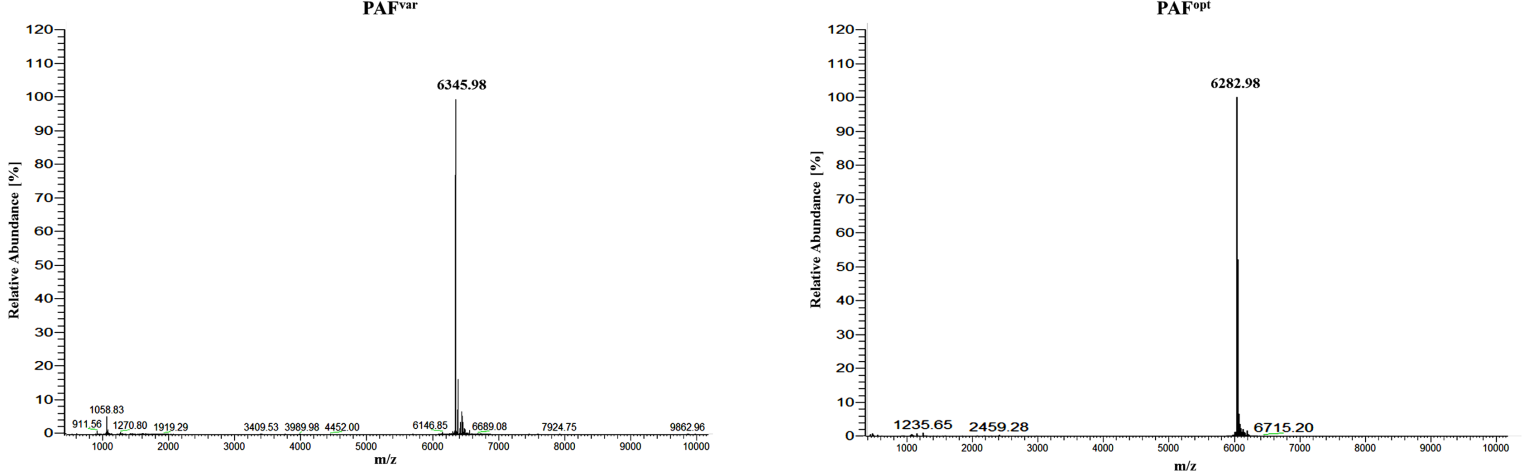


**Figure S5.** **Mass spectrometry of PAF variants**. ESI-MS proved the correct amino acid exchange and processing of PAF^var^ (6,345.98 Da) and PAF^opt^ (6,282.98 Da). No impurities could be detected.

**
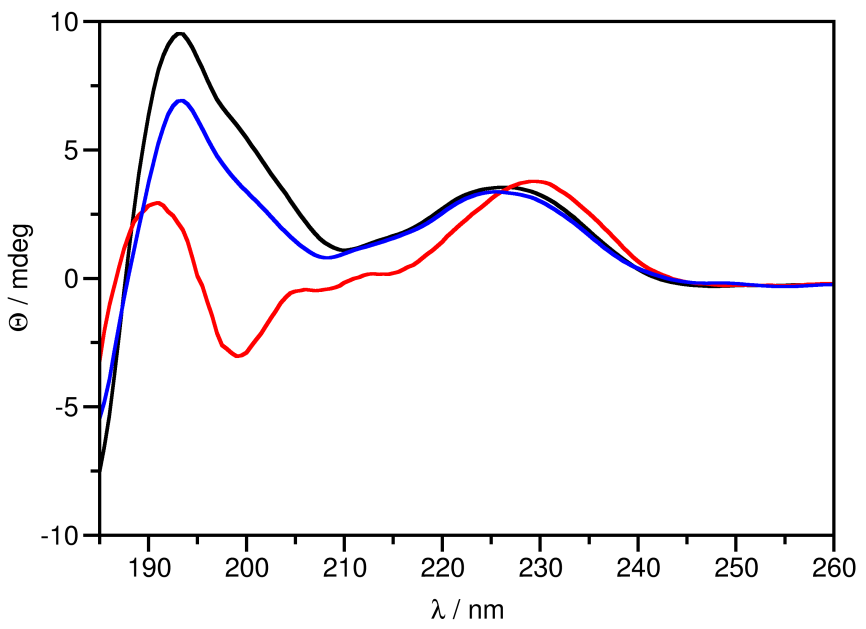
**

**Figure S6.** **ECD spectra of PAF variants.** Comparative overlay of ECD spectra of PAF (black) (Sonderegger et al., 2016), PAF^var^ (red) and PAF^opt^ (blue), acquired in pure H_2_O at 25 °C.


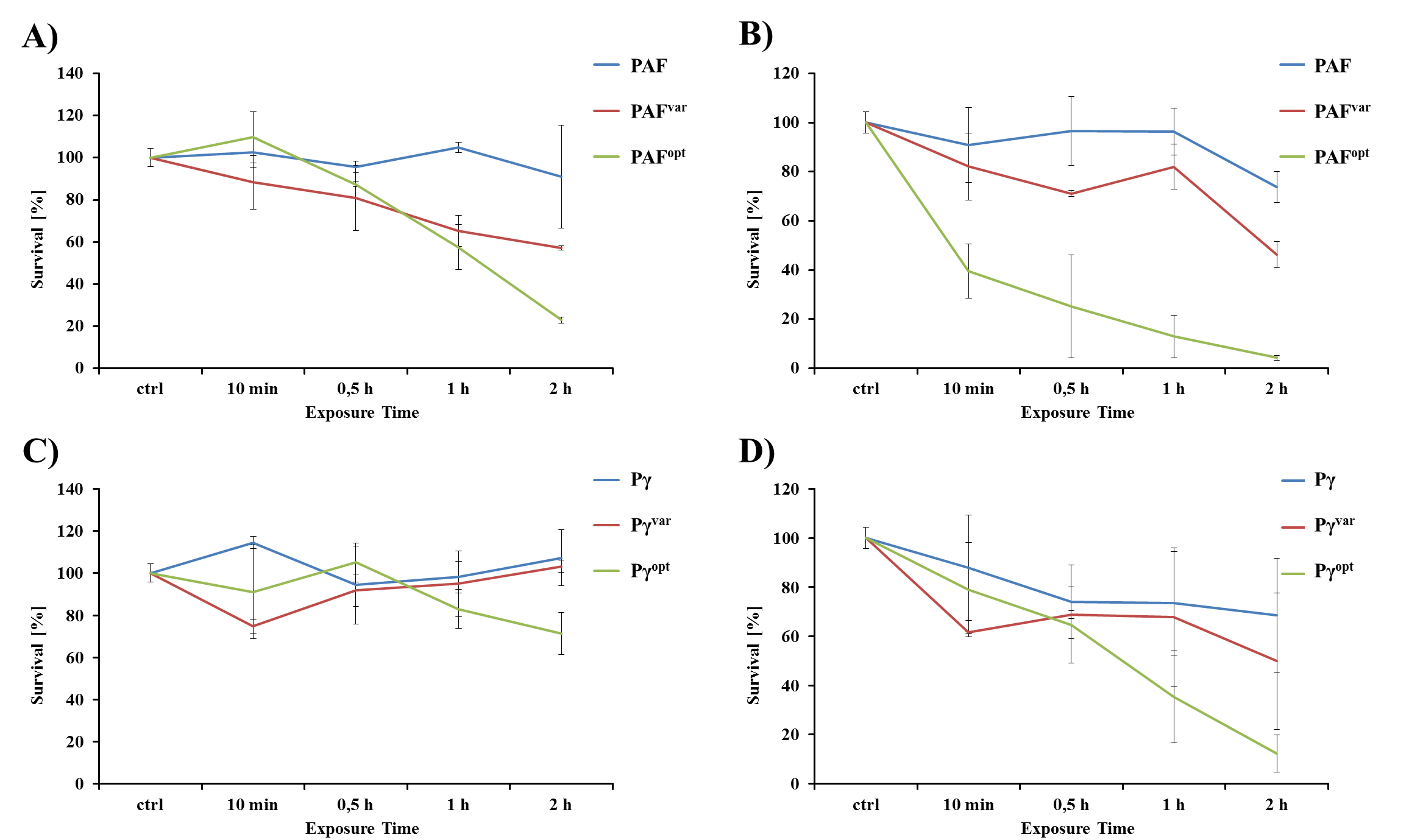


**Figure S7. Fungicidal activity of AMPs.** *C. albicans* was exposed to **(A, C)** 1x MIC and **(B, D)** 2x MIC of AMPs before plating appropriate dilutions on agar plates to determine viable cfu. The untreated control was set as 100%. Values represent mean ± SD.


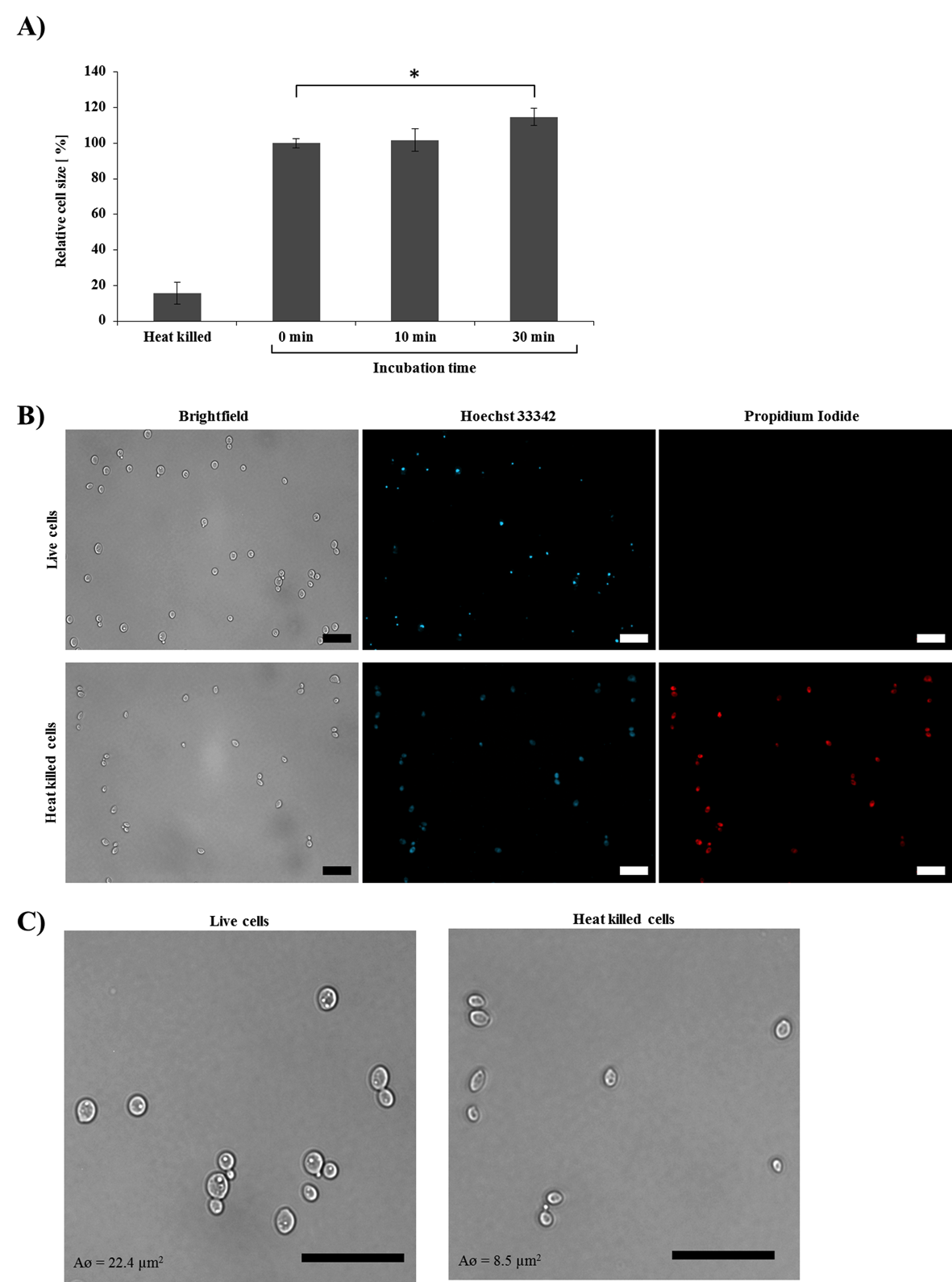


**Figure S8. Cell size of *C. albicans*.** **(A)** Yeast cells were maintained in growth medium for up to 30 minutes and cell size was analyzed by flow cytometry (% change in FSC) at the indicated time points. Cells at 0 minutes were set as 100% and heat inactivated cells were used as a control. Values represent mean ± SD; * *P* = 0.045. **(B)** Images were taken from the samples in **(A)** to visualize cell death after heat-inactivation of *C. albicans*. Nuclei were stained with Hoechst 33342 and dead cells with propidium iodide. **(C)** Magnified section of the brightfield image in **(B)** that illustrates the reduced cell size of dead *C. albicans* cells. The average cell area (Aø) of the displayed cells was measured with ImageJ (Schindelin et al., 2012). Scale bars = 20 µm.


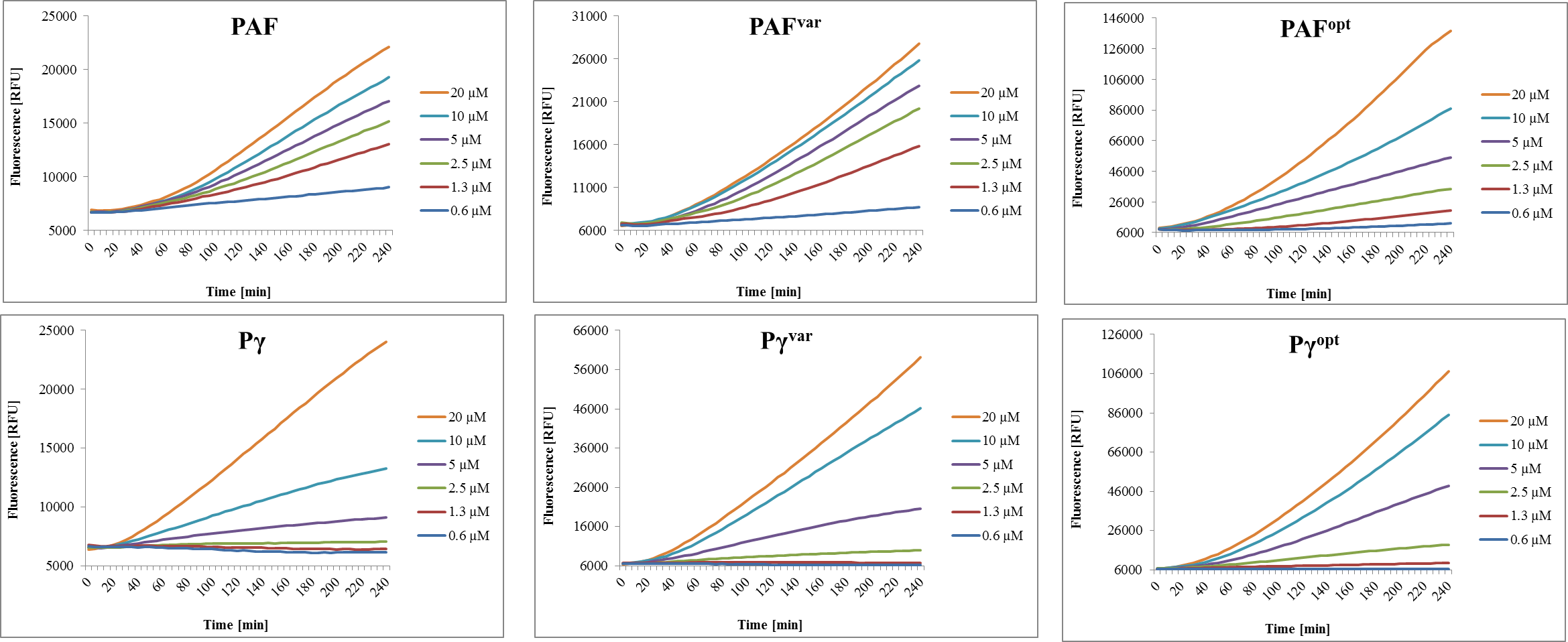


**Figure S9. ROS production in *C. albicans* exposed to AMPs in time-course.** Fungal cells were pre-treated with DCFH-DA and exposed to AMPs ranging from 0–20 µM. DCF fluorescence intensities (RFU) were detected every five minutes over a four-hour time course. Values represent the mean from three independent experiments.


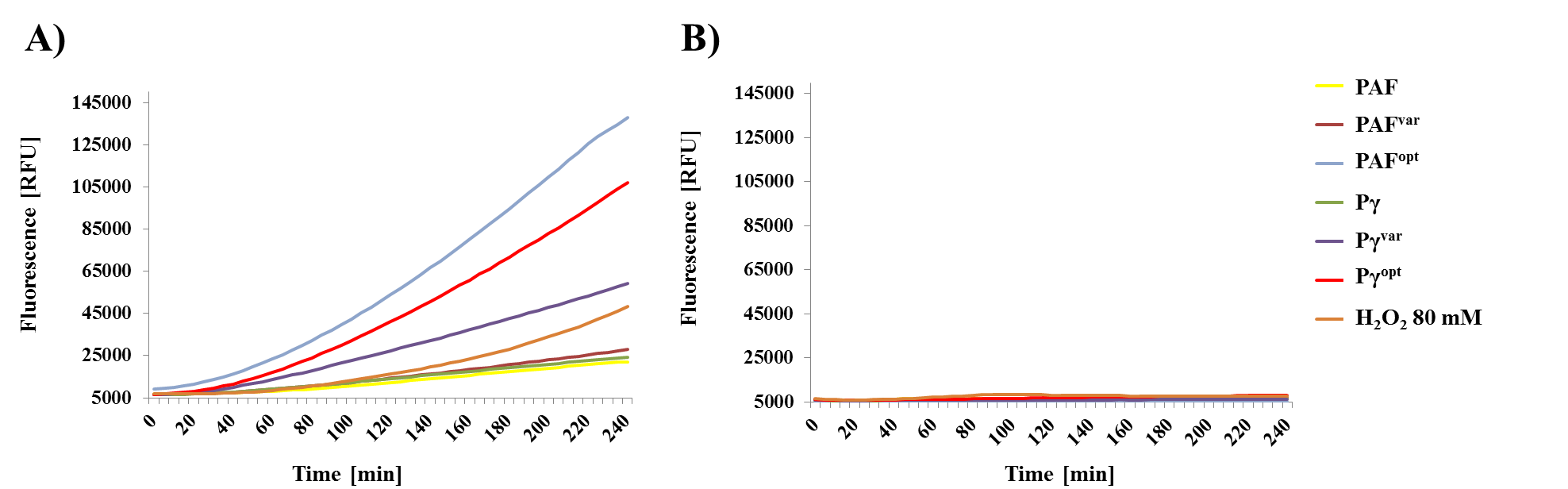


**Figure S10. AMP-dependent ROS production in *C. albicans* is prevented in the presence of Vitamin C.** Fungal cells were exposed to **(A)** 20 µM AMPs and **(B)** 20 µM AMPs plus 200 µg mL^‑1^ Vitamin C for up to four hours and DCF fluorescence intensities were detected every five minutes.


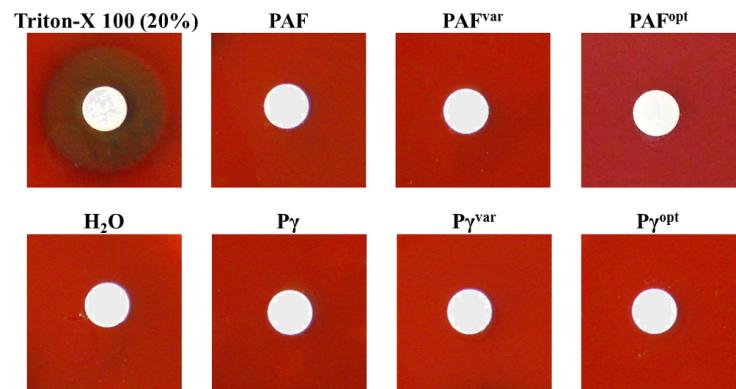


**Figure S11.** **Hemolytic activities of AMPs.** Paper filter discs (Ø 5 mm) were put on Columbia blood agar and soaked in 15 µL (20 µg) of each AMP. The agar plates were incubated for 24 hours at 37 °C, including positive (20% Triton-X 100) and negative controls (H_2_O).


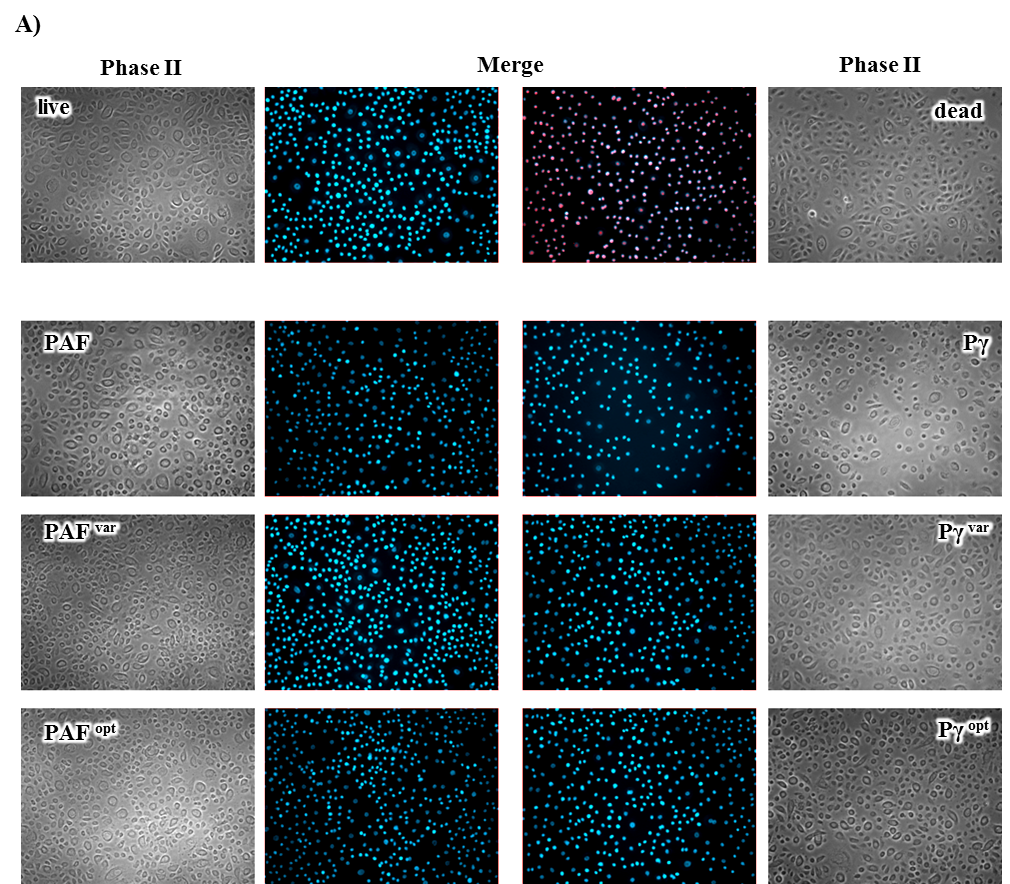


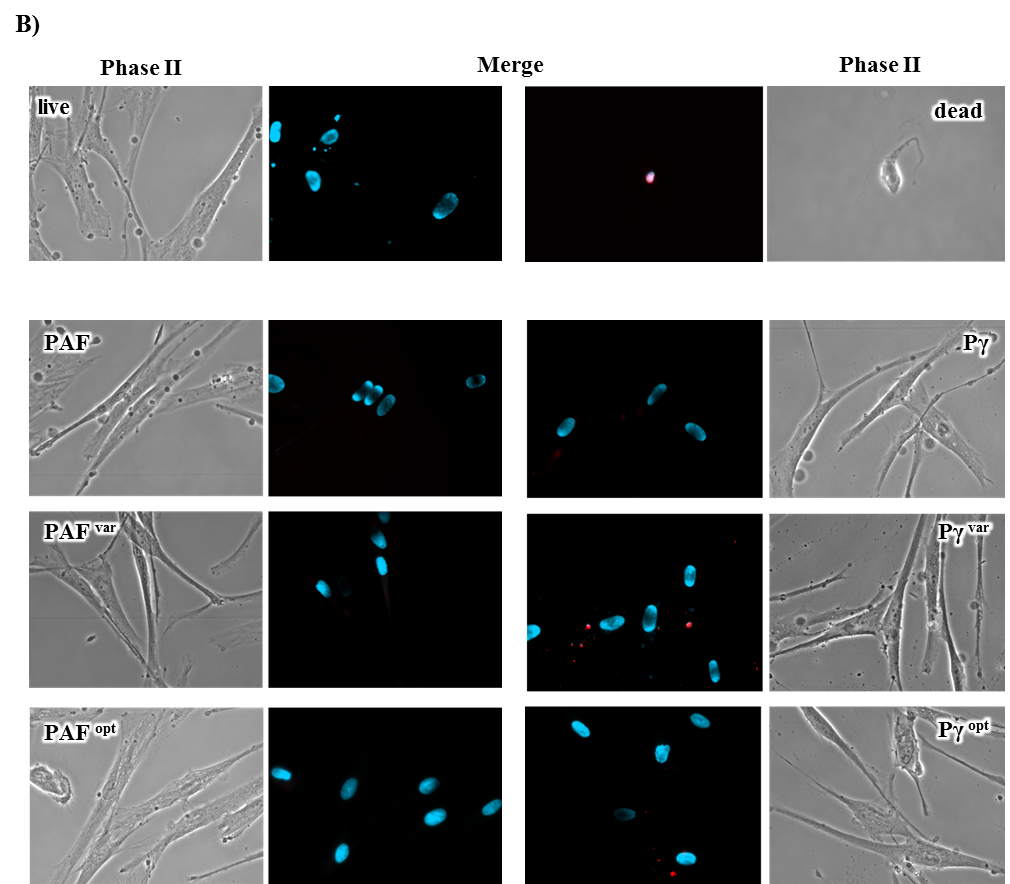


**Figure S12. Toxicity-testing of AMPs against primary human skin cells.** Fluorescence staining with propidium iodide (red) and Hoechst 33342 (blue) of **(A)** keratinocytes and **(B)** fibroblasts after 24 hours exposure to 30 µM AMPs. Controls: "live" cells remained untreated and "dead" cells were ethanol treated.


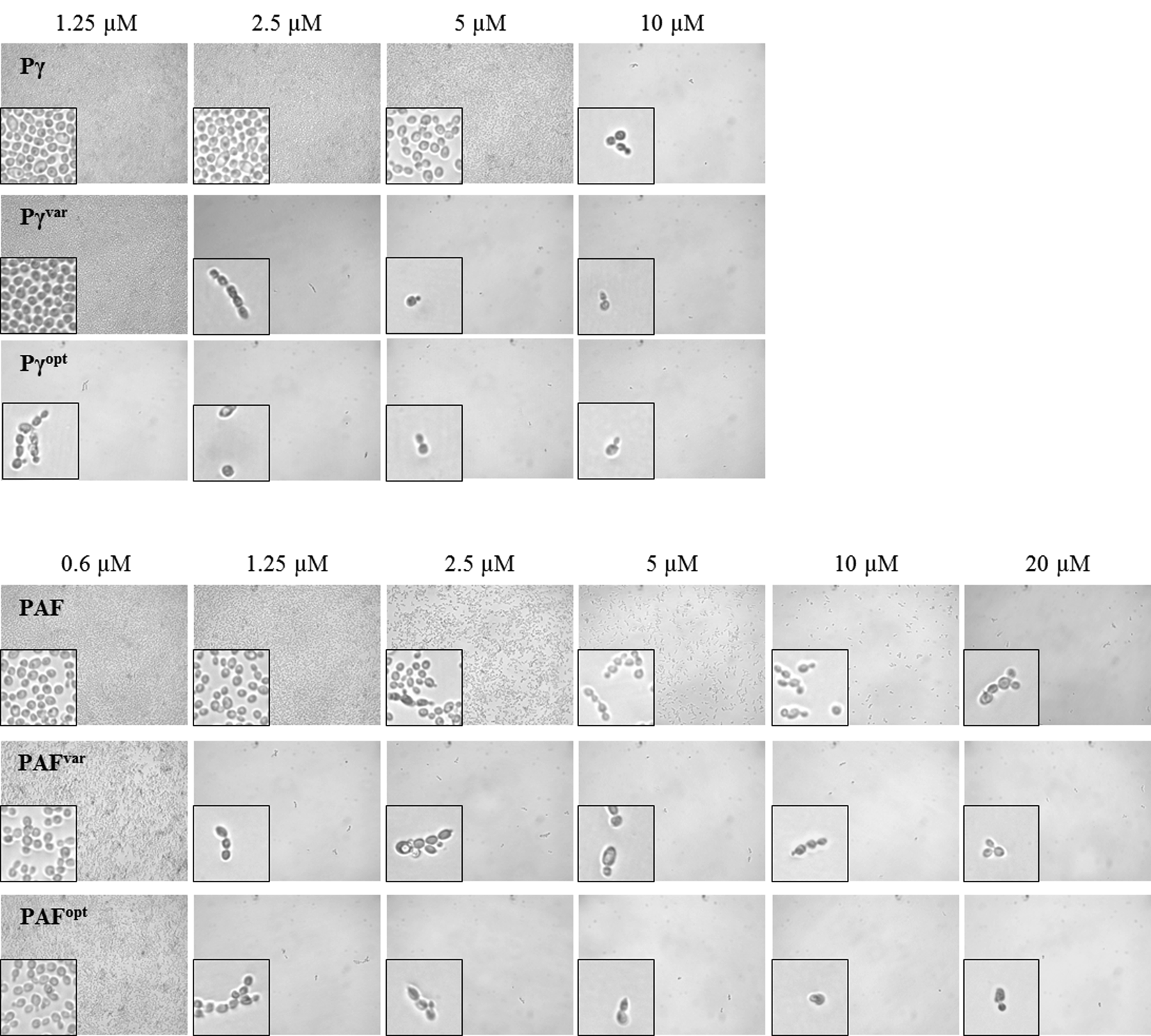


**Figure S13.** **Growth reduction and cell morphology of AMP treated *C. albicans*.** Fungal cells were exposed to increasing concentrations of AMPs (upper panel: peptides; lower panel: proteins) and microscopic images were taken after 24 hours of incubation. The insets are magnifications of the respective overview images to visualize the morphology of *C. albicans* cells.

Table S1. **Fungal strains used in this study.**

| **Strain** | **Genotype** | **Reference** |
| --- | --- | --- |
| *Candida albicans* | wild-type, human isolate | CBS 5982 |
| *Penicillium chrysogenum* *∆paf* | *∆paf*:*nat1* | (Hegedüs et al., 2011) |
| *P. chrysogenum paf* | *∆paf*:*nat1, paf*^+^, *ptrA*^+^ | (Sonderegger et al., 2016) |
| *P. chrysogenum* *paf*^T8Y S10K^ | *∆paf*:*nat1, paf*^T8Y S10K^, *ptrA*^+^ | this study |
| *P. chrysogenum* *paf* ^T8K K9T S10K E13K^ | *∆paf*:*nat1, paf* ^T8K K9T S10K E13K^, *ptrA*^+^ | this study |

**Table S2. Composition of media used in this study.**

| **Description** | **Composition** |
| --- | --- |
| *P. chrysogenum* minimal medium (*Pc*MM) | 2% sucrose, 0.3% NaNO_3_, 0.05% KCl, 0.05% MgSO_4_ x 7 H_2_O, 0.005% FeSO_4_ x 7 H_2_O, 0.1% trace elements solution, 25 mM NaCl/P_i_ (pH 5.8) |
| Trace elements solution | 0.1% FeSO_4_ x 7 H_2_O, 0.9% ZnSO_4_ x 7 H_2_O, 0.4% CuSO_4_ x 5 H_2_O, 0.01% MnSO_4_ x 1 H_2_O, 0.01% H_3_BO_3_, 0.01% Na_2_MoO_4_ x 2 H_2_O |
| R10 (Fibroblast cell culture medium) | RPMI 1640 (Lonza BE12-167F), 10 mM HEPES buffer (Biochrom-Merck L1613), 0.1% gentamicin (Gibco 15750-037), 10% heat inactivated FCS (PAN Biotech P30-1502), 2 mM L-alanyl-L-glutamine (GlutaMAX, Gibco 35050-038) |
| YPD (Yeast extract peptone dextrose medium) | 1% yeast extract (Lab M), 2% bacteriological peptone (Lab M), 2% D-(+)-glucose (Carl Roth) |

Table S3. **Oligonucleotides used in this study.** Mutation primers for generation of PAF variants are in bold and mismatches for amino acid substitutions are underlined.

| **Name** | **Sequence 5'-3'** |
| --- | --- |
| M13 | GTAAAACGACGGCCAGTGAG |
| T7var | TACGACTCACTATAGGGCG |
| opaf10 | GCTGCCACCCCCAAGATGACTG |
| opaf11 | CACTCCCCTCATACTTCATG |
| opaf12 | CTTCTCTGACTGAAAGTACC |
| opaf14 | CCCAATTTAATGCGGTCCTGC |
| opaf15 | GCCTCTTCGCTATTACGCC |
| opaf^T8Y S10K_fw^ | CAGAAATGCTACAAAAAGAAGAAC |
| opaf^T8Y S10K_rev^ | GATGTTCTTCTTTTTGTAGCATTTC |
| opaf^T8K K9T S10K E13K_fw^ | CAGAAATGCAAGACCAAGAAGAACAAGTGTAAATAC |
| opaf^T8K K9T S10K E13K_rev^ | GTATTTACACTTGTTCTTCTTGGTCTTGCATTTCTG |

**Table S4.** **Sequence characteristics of the γ-core peptides based on their physicochemical features.** The analysis was performed with the online analysis tool ([http://www.biosyn.com/peptidepropertycalculatorlanding.aspx#](http://www.biosyn.com/peptidepropertycalculatorlanding.aspx)).

|  |  | **Hydrophobicity/Hydrophilicity analysis in %** | | | |
| --- | --- | --- | --- | --- | --- |
| **Peptide name** | **Peptide sequence** | **Hydrophobic** | **Acidic** | **Basic** | **Neutral** |
| Pγ | KYTGKCTKSKNECK | 21.4 | 7.1 | 35.7 | 35.7 |
| Pγ^var^ | KYTGKCYKKKNECK | 28.6 | 7.1 | 42.9 | 21.4 |
| Pγ^opt^ | KYTGKCKTKKNKCK | 21.4 | 0 | 50 | 28.6 |

**Table S5. ECD spectrum deconvolution for PAF and PAF^opt^.**

|  | **Helix1** | **Helix2** | **Strand1** | **Strand2** | **Turns** | **Unordered** | **Total** |
| --- | --- | --- | --- | --- | --- | --- | --- |
| **PAF** | 0.00 | 0.04 | 0.39 | 0.21 | 0.15 | 0.21 | 1.00 |
| **PAF^opt^** | -0.10 | 0.02 | 0.27 | 0.14 | 0.20 | 0.36 | 0.98 |

**Table S6.** **Putative AMP homologs of Eurotiomycetes from MycoCosm portal (Grigoriev et al., 2014) of DOE Joint Genome Institute.** Red letters indicate the putative prepro-sequence and bold letters the mature protein. The γ-core motifs are highlighted with grey background. White, red, and blue letters indicate neutral (-0.2 – +0.3 at pH 7.0), negatively and positively charged γ-cores respectively. * The same isolate.

| **Fungus** | **Protein ID\|Model name** | **Net charge of γ-core(s) at pH=7.0** | **GRAVY of γ-core(s)** |
| --- | --- | --- | --- |
| **PAF-cluster proteins** | | | |
| MQITSIAIVLFAAMGAIANPIAAEADDLLAREAQ**LSKYGGECSLEHNTCTYRKDGKNHVVACPTAANLRCKTDRHHCEYDDHHKTVDCQTPV** | | | |
| *Aspergillus brasiliensis* | 108415\|gw1.5.1853.1 | -1.9 | -0.68 |
| MQISSISIVLFAAMGAVAKPFAAGSDDLNARDIQ**LSKYGGECSLKHNTCTYRKGGKNQIVKCGSAANKKCKSDRHHCEYDEHHKTVNCQTPV** | | | |
| *Aspergillus brevijanus* CBS 111.46 | 277361\|MIX991_1_54 | +0.1 | -0.72 |
| MQITSIAIVLFAAMGAVANPIATESDNLDARDVQ**LSKFGGECSLKHNTCTYLKGGKNHVVNCGSAANKKCKSDRHHCEYDEHHKTVDCQTPV** | | | |
| *Aspergillus chevalieri* CBS 522.65 v1.0 | 199501\|gm1.9097_g | +0.1 | -0.72 |
| MQLTNIAIILFAAMGAIANPIAAESDDLLARDVQ**LSKYGGECSLEHNTCTYRKDGKNHVVSCPSAANLRCKTDRHHCEYDDHHKTVDCQTPV** | | | |
| *Aspergillus costaricaensis* CBS 115574 | 286799\|estExt_Genemark1.C_120084 | -1.9 | -0.68 |
| MQLTNIAIILFAAMGAIANPIAAESDDLLARDAQ**LSKYGGECSLEHNTCTYRKDGKNHVVSCPSATNKRCKTDRHHCEYDDHHKTVDCQTPV** | | | |
| *Aspergillus kawachii* IFO 4308 | 15612\|AKAW_04140m.01 | -1.9 | -0.68 |
| MQLTSIAIILFAAMGAIANPIAAESDDLLARDAQ**LSKYGGECSLEHNTCTYRKDGKNHVVSCPSATNLRCKTDRHHCEYDDHHKTVDCQTPV** | | | |
| *Aspergillus luchuensis* CBS 106.47 | 214633\|estExt_Genemark1.C_2_t10089 | -1.9 | -0.68 |
| MQLTSIAIILFAAMGAIANPIAAEADNLVAREAE**LSKYGGECSVEHNTCTYLKGGKDHIVSCPSAANLRCKTERHHCEYDEHHKTVDCQTPV** | | | |
| *Aspergillus lacticoffeatus* CBS 101883 | 440952\|gm1.729_g | -1.9 | -0.64 |
| MQLTSIAIILFAAMGAIANPITAESDDLLARDAQ**LSKYGGECSLEHNTCTYRKDGKNHVVSCPSATNLRCKTDRHHCEYDDHHKTVDCQTPV** | | | |
| *Aspergillus neoniger* CBS 115656 | 379350\|fgenesh1_kg.32_*_23_*_Locus4v1rpkm28984.84 | -1.9 | -0.68 |
| MQLTSIAIILFAAMGAIANPIAAEADNLVAREAE**LSKYGGECSVEHNTCTYLKGGKDHIVSCPSAANLRCKTERHHCEYDEHHKTVDCQTPV** | | | |
| *Aspergillus niger* CBS 513.88 | 160366\|An07g01320m.01 | -1.9 | -0.64 |
| MQLTSIAIILFAAMGAIATPITAEADNLAAREAE**LSKYGGECSVEHNTCTYLKGGKDHIVSCPSAANLRCKTERHHCEYDEHHKTVDCQTPV** | | | |
| *Aspergillus niger* NRRL3 | 4330\|NRRL3_04330 | -1.9 | -0.64 |
| MKLTSIAIILFAAMGAIANPIAAESDDLLARDVQ**LSKYGGECSLEHNTCTYRKDGKNHVVSCPSATNLRCKTDRHHCEYDDHHKTVDCQTPV** | | | |
| *Aspergillus eucalypticola* CBS 122712 | 383499\|fgenesh1_kg.46_*_38_*_Locus15133v1rpkm0.70 | -1.9 | -0.68 |
| MQLTSIAIILFAAMGAIANPITAEANNLVAREEE**LSKYGGECSVEHNTCTYLKGGKDHIVSCPSAANLRCKTERHHCEYDEHHKTVDCQTPV** | | | |
| *Aspergillus phoenicis* ATCC 13157 | 339122\|estExt_Genemark1.C_230094 | -1.9 | -0.64 |
| MQLTSIAIILFAAMGAIANPIATESDDLLARDAQ**LSKYGGECSLEHNTCTYRKDGKNHVVSCPSATNLRCKTDRHHCEYDDHHKTVDCQTPV** | | | |
| *Aspergillus piperis* CBS 112811 | 465083\|estExt_fgenesh1_pm.C_1_t20013 | -1.9 | -0.68 |
| MQLTSIAIILFAAMGAIANPIAAESDDLLARDAQ**LSKYGGECSLEHNTCTYRKDGKNHVVSCPSAANLRCKTDRHHCEYDDHHKTVDCQTPV** | | | |
| *Aspergillus tubingensis* | 202739\|estExt_Genemark1.C_6_t10079 | -1.9 | -0.68 |
| MQLTSIAIILFAAMGAIANPIATESDDLLARDAQ**LSKYGGECSLEHNTCTYRKDGKNHVVSCPSATNLRCKTDRHHCEYDDHHKTVDCQTPV** | | | |
| *Aspergillus vadensis* CBS 113365 | 455414\|gm1.7532_g | -1.9 | -0.68 |
| MQLTSIAIILFAAMGAIATPITAEADNLVAREAE**LSKYGGECSVEHNTCTYLKGGKDHIVSCPSAANLRCKTERHHCEYDEHHKTVDCQTPV** | | | |
| *Aspergillus welwitschiae* CBS 139.54b | 172455\|gm1.7704_g | -1.9 | -0.64 |
| MQFTKIAIFLFAAMGAVANPIAAESGDLDVRDVQ**LSKYGGECSLQHNTCTYLKGGKNQVVHCGSAANQKCKSDRHHCEYDEHHKTVNCQTPV** | | | |
| *Monascus ruber* NRRL 1597 | 434904\|fgenesh1_kg.74_*_59_*_Locus401v1rpkm368.85 | -0.9 | -0.68 |
| MQITKISLFLFVGIGVVASPIHAESDGLNARAVNAAD**LEYKGECFTKDNTCKYKIDGKTYLAKCPSAANTKCEKDGNKCTYDSYNRKVKCDFRH** | | | |
| *Neosartorya fischeri* NRRL 181 | 4785\|7000001156984285 | -1.1 | -0.84 |
| MQITKISLFLFAAIAAVANPIDAESDGIVERDVDAAD**ITYTGQCFRKNNECRYVANGKTHYVKCPSKFANKRCQMDKHKCTFDSYSRVVNCNA** | | | |
| *Paecilomyces variotii* CBS 101075 | 453640\|fgenesh1_kg.1_*_4840_*_TRINITY_DN12373_c0_g2_i1 | +0.8 | -1.50 |
| MQIISIAIVLFAAMGAVATPIATESDDLDARDVQ**LSKYGGECSLKHNTCTYRKDGKDHVVNCGSATNRKCKTDRHHCEYDDHHKTVDCQTPV** | | | |
| *Penicillium antarcticum* IBT 31811 | 2600\|PENANT_c015G07856T0 | +0.1 | -0.72 |
| MHITSIAIVFFAAMGAVASPIATESDDLDARDVQ**LSKFGGECSLKHNTCTYLKGGKNHVVNCGSAANKKCKSDRHHCEYDEHHKRVDCQTPV** | | | |
| *Penicillium chrysogenum* Wisconsin 54-1255 | 137905\|PCH_Pc12g08290 | +0.1 | -0.72 |
| MQITTVALFLFAAMGGVATPIESVSNDLDARAEAGVL**AKYTGKCTKSKNECKYKNDAGKDTFIKCPKFDNKKCTKDNNKCTVDTYNNAVDCD** | | | |
| *Penicillium chrysogenum* Wisconsin 54-1255 | 150051\|PCH_Pc24g00380 | +1.8 | -1.56 |
| MQITKVALFLFAAMGAMATPIESVENGLDARAEAGVL**AKYTGTCNRAKNECKYKNDRGKTTFIKCPSKIANKRCTKDGAKCTVDTYNNSVDCD** | | | |
| *Penicillium coprophilum* IBT 31321 | 2359\|PENCOP_c011G02314T0 | +0.8 | -1.32 |
| MQITSIAIILFTAMGAVANPIATASDDLDARDVQ**LSKYGGQCSLKHNTCTYLKGGRNVIVNCGSAANKRCKSDRHHCEYDEHHRRVDCQTPV** | | | |
| *Penicillium digitatum* PHI26 | 6476\|PDIG_68840m.01 | +1.1 | -0.72 |
| MQITRIAIFLFAAMGAVASPIVAESRDVDAQA**LSKYGGECSKEHNTCTYRKDGKDHIVKCPSADNKKCKTDRHHCEYDGHHKTVDCQTPV** | | | |
| *Penicillium expansum* ATCC 24692 | 444546\|estExt_Genemark1.C_6_t10265 | -0.9 | -1.45 |
| MQITKIALFLFAAMGAVASPIEAEAESGINARAENGAN**VLYTGQCFKKDNICKYKVNGKQNIAKCPSAANKRCEKDKNKCTFDSYDRKVTCDFRK** | | | |
| *Penicillium expansum* ATCC 24692 | 376451\|fgenesh1_kg.4_*_1163_*_Locus5213v1rpkm9.21 | +0.8 | -0.64 |
| MQITRIAIFFFAAMGAVANPITNDLNAQA**LSKYGGECSKEHNTCTYRKDGKDHKVKCPSADNLKCKTDRHHCEYDDHHKKVDCQTPV** | | | |
| *Penicillium italicum* PHI-1 | 9261\|PITC_075430T0 | -0.9 | -1.45 |
| MQITSIAIVLFAAMGAVANPIPTESDDLVARDVQ**LSKFGGECSLKHNTCSYRKGGKTRIVNCGSAANKKCKTDRHHCEYDEHHRRVDCQTPV** | | | |
| *Penicillium polonicum* IBT 4502 | 10100\|PENPOL_c008G10355T0 | +0.1 | -0.72 |
| MQITSIAIALFAAMVVVANPIATDSDSLGARDAQ**LSKYGGECSLQHNTCTYRKDGKNHVVNCPTATNKKCKTDRHHCEYDDHHKTVDCQTPV** | | | |
| *Penicillium raistrickii* ATCC 10490 | 364170\|gm1.10905_g | -0.9 | -0.68 |
| MQITSIAIVLFAAMGAVANPTATESDGLDARDVE**LSKYGGECSLAHNTCTYLKGGKNQVVACGTAANKRCKTDRHHCEYDEYHKMVDCQTPV** | | | |
| *Penicillium subrubescens* FBCC1632 / CBS132785 | 1094\|scaffold_157.8 | -0.9 | -0.15 |
| MQITTVALFLFAAMGAVATPIESVSNGLDARAEAGIL**AKYTGKCTKSKNECKYKNDAGKDTFIKCPKFDNKKCTKDGNKCTVDTYNNAVDCD** | | | |
| *Penicillium swiecickii* 182 6C1 v1.0 | 362586\|estExt_fgenesh1_pm.C_140186 | +1.8 | -1.56 |
| MQITRIAIVLFAAMGAVANPVATESNDLDAEAF**GSKYGGECSKQHNTCKYRKNGKTHIIKCPSANNLKCKTDRHHCEYDEHHKKVDCQTPV** | | | |
| *Penicillium vulpinum* IBT 29486 | 95\|PENVUL_c001G01660T0 | +0.1 | -1.45 |
| **AFP-cluster proteins** | | | |
| MRISPVSISFIILAAMGVAATPLNHAESVGVRSENNVQ**VKYDGQCRKSENQCRYTAQSGRTAICKCQFRKCSKDGAKCNFDSYNRDCNCY** | | | |
| *Aspergillus aculeatinus* CBS 121060 | 471682\|estExt_Genemark1.C_260034 | +0.8 | -1.86 |
| MKISPVSISFIILAAMGVAATPLNHAESVGVRSENNVQ**VKYDGQCRKSENQCRYTAQSGRTAICKCQFRKCSKDGAKCNFDSYNRDCNCY** | | | |
| *Aspergillus brunneoviolaceus* CBS 621.78 | 277955\|CE277954_62 | +0.8 | -1.86 |
| MQLISLASMGLVLFAAVGAVASPVDNNALDVNDNLEVHDEAATL**ITYNGSCSKKNNSCKYKGQKGKTSFCHCKFKKCGKDGNKCHFDSYSRDCKCI** | | | |
| *Aspergillus campestris* IBT 28561 | 329071\|estExt_Genemark1.C_10_t10236 | +1.8 | -1.26 |
| MQLISLASMGLVLFAAVGAVASPMDNNALDVNDNLEVRDEAATL**IKYHGHCSKKNNSCKFKGQHGKTSFCHCKFKKCSRDGNKCHFDSYSRDCKCI** | | | |
| *Aspergillus candidus* CBS 102.13 | 104765\|fgenesh1_kg.18_*_257_*_TRINITY_DN6146_c0_g1_i1 | +2.1 | -1.50 |
| MKMSPVSISFIILAAMGVAATPLNHAESVGVRSENNVQ**VKYDGQCRKSENQCRYTAQSGRTAICKCQFRKCSKDGAKCNFDSYNRDCNCY** | | | |
| *Aspergillus fijiensis* CBS 313.89 | 309816\|CE309815_48 | +0.8 | -1.86 |
| MKISPVSISFIILAAMGVAATPLNHAESVGVRSENNVQ**VKYDGQCRKSENQCRYTAQSGRTAICKCQFRKCSKDGAKCNFDSYNRDCNCY** | | | |
| *Aspergillus indologenus* CBS 114.80 | 430342\|fgenesh1_kg.134_*_18_*_Locus199v2rpkm1.21_PRE | +0.8 | -1.86 |
| MKISPVSIGFILLAAMGVAATPLNHAESVGVRSENNVQ**VKYDGQCRKSENQCRYTAQSGRTAICKCQFRKCSKDGAKCNFDSYNRDCNCY** | | | |
| *Aspergillus japonicus* CBS 114.51 | 129218\|CE129217_19 | +0.8 | -1.86 |
| MQLISLASMGLVLFAAVGAVASPVDNNALDIDNNLEVRDEAASL**IKYHGVCSKKNNSCKFKGQNGKTSFCHCKFKKCGKENNKCHFDSYNRDCKCI** | | | |
| *Aspergillus taichungensis* IBT 19404 | 192584\|gm1.2851_g | +1.8 | -0.76 |
| MKISPVPISFIILAAMGVAASPLDHAEAVGVRSENNVQ**VKYDGQCRKSQNQCRYTAQSGRTAICKCQFRKCSKDGARCNFDSYNNDCNCY** | | | |
| *Aspergillus uvarum* CBS 121591 | 402664\|gm1.5743_g | +1.8 | -1.86 |
|  |  |  |  |
| **BP-cluster proteins** | | | |
| MKLTALLCTLMAAAAVSASPVLEAR**DTCGAGYSGDQRRTNSACEASNGDRDFCGCDRTGVVQCQGGIWTEIEDCGSATCAGGDQGGAAC** | | | |
| *Aspergillus amylovorus* CBS 600.67 | 165232\|gm1.9571_g | -0.1 / -0.2 | -0.28 / +0.79 |
| MKATTIFYTLLAATAVSASAVPQNDFEIL**DTCGAGYGGDQRRTNSPCASSNGDRHFCGCDRTGVVQCKGGKWTEIQDCHSGTCHGGNDGGAVC** | | | |
| *Aspergillus bombycis* NRRL 26010 | 2197\|ABOM_011038T0 | +0.8 / +0.3 | -0.32 / -0.46 |
| MKLIAIVCTLMAAAAVSASTIEAR**DTCGAGYGGDQRRTNSPCAASNGDRHFCGCDRTGVVECKGGKWTEVKDCGRGTCHGGNQGAAQC** | | | |
| *Aspergillus campestris* IBT 28561 | 318976\|gm1.5151_g | -0.1 / +1.1 | -0.32 / -0.58 |
| MKLIAIVCTLMAAASVSASTIEAR**DTCGAGYGGDQRRTNSPCASSNGDRHFCGCDRTGIVECKGGKWTEVKDCGSGTCHGGNQGAAQC** | | | |
| *Aspergillus candidus* CBS 102.13 | 105744\|fgenesh1_kg.22_*_137_*_TRINITY_DN7228_c0_g1_i1 | -0.1 / +0.1 | -0.29 / -0.11 |
| MKFTALLCTLMAATAVSASTLPRGEFQVQ**DTCGAGYGGDQRRTNSPCNASNGDRHFCGCDRTGVVECRGGKWTEIQDCHASTCHGTNDGAARC** | | | |
| *Aspergillus novofumigatus* IBT 16806 | 459645\|fgenesh1_pm.3_*_1203 | -0.1 / +0.3 | -0.37 / -0.19 |
| MKLIAIVCTLMAAASVSASNIQAR**DTCGAGYGGDQRRTNSPCAASNGDRHFCGCDRTGIVECKGGKWTEVKDCGSGTCHGGNQGAAQC** | | | |
| *Aspergillus taichungensis* IBT 19404 | 143461\|CE143460_23868 | -0.1 / +0.1 | -0.29 / -0.11 |
| MKLIAIFTTLMAAAAVSASTIEAR**DTCGAGYGGDQRRTNSPCAASNGDRHFCGCDRTGIVECKGGKWTEVKDCGSGTCHGGNQGAAQC** | | | |
| *Aspergillus triticus* CBS266.81 | 134014\|gm1.5780_g | -0.1 / +0.1 | -0.29 / -0.11 |
| MKFTSLILTLMAAAAVTAAPSPELEVR**DTCGPGYGGDQRRTNSPCNASNGDRHFCGCDRTGVVECRGGRWTEIRDCGRGTCHGGNDGGAVC** | | | |
| *Aspergillus versicolor** | 79906\|gm1.2374_g | -0.1 / +1.1 | -0.37 / -0.58 |
| MKLSIFFATLLAAAVSAGSVLEAR**DTCGAGYGGDQRRTNSACDASNGDRHFCGCDRTGVVECQGGTWTEISDCGSGTCHGGNDGGAQC** | | | |
| *Aspergillus versicolor** | 85611\|gm1.8079_g | -1.1 / +0.1 | -0.28 / -0.11 |
| MKFTALLCTLMAATAVSASTVPRDEFQIQ**DTCGAGYGGDQRRTNSPCNASNGDRHFCGCDRTGVVECRGGRWTEIQDCRASTCHGTNDGAARC** | | | |
| *Neosartorya fischeri* NRRL 181 | Neofi1\|9128\|7000001157005755 | -0.1 / +1.1 | -0.37 / -0.35 |
| MKFTALLFTLMAAATVSASPADDPQIL**DVCGSGYGGDQRRTNSGCQSSNGDRHFCGCDRTGVVECKGGKWTEIQDCHKSSCHGSNQGGAVC** | | | |
| *Penicillium antarcticum* IBT 31811 | 9008\|PENANT_c005G04226T0 | -0.1 / +1.3 | -0.32 / -0.91 |
| MKITALLYTLTAATAVSAAAVAERDTLGGLEAR**DTCGSGYNVDQRRTNSGCKAGNGDRHFCGCDRTGVVECKGGKWTEVQDCGSSSCKGTSNGGATC** | | | |
| *Penicillium brevicompactum* 1011305 | 53502\|gm1.665_g | -0.1 / +0.8 | -0.32 / -0.26 |
| MKITALLYTLMAATAVSAAAVAERDTLGGLEAR**DTCGSGYNVDQRRTNSGCKAGNGDRHFCGCDRTGVVECKGGKWTEVQDCGSSSCKGTSNGGATC** | | | |
| *Penicillium brevicompactum* AgRF18 | 348372\|gm1.2484_g | -0.1 / +0.8 | -0.32 / -0.26 |
| MKVTALLFTLMAATAVSASVLDTR**DTCGGGYGVDQRRTNSPCQASNGDRHFCGCDRTGIVECKGGKWTEIQDCGGASCRGVSQGGARC** | | | |
| *Penicillium chrysogenum* Wisconsin 54-1255 | 146100\|PCH_Pc21g12970 | -0.1 / +0.8 | -0.29 / -0.04 |
| MKFTAMLFTLMAATAVSASVLEAR**DGCGSAYDPDQRRTNSPCQNSNGNKQYCGCDRSGIVQCKGGKWTEVRDCGNSPCHGGKEGGALC** | | | |
| *Penicillium coprophilum* IBT 31321 | 7078\|PENCOP_c006G07664T0 | +0.8 / +0.1 | -0.30 / -0.61 |
| MKVTAILFTLMAATAVSASVLDKR**DTCGAGYDPAQRRTNSPCQASNGDRHFCGCDRTGIVECKGGKWTEIQDCGRNSCHGGTEGGAKC** | | | |
| *Penicillium expansum* ATCC 24692 | 441362\|estExt_Genemark1.C_3_t40302 | -0.1 / +1.1 | -0.29 / -0.98 |
| MKVTALLFTLMAATAVSASVLETR**DTCGSGYGVDQRRTNSPCQASNGDRRFCGCDRTGVVQCKGGKWTEVQDCGRGSCRGGNQGGAKC** | | | |
| *Penicillium flavigenum* IBT 14082 | 9096\|PENFLA_c006G04053T0 | +0.8 / +1.8 | -0.32 / -0.75 |
| MKVTSFFLTIMAATAATASVLNTR**DTCGSGYAPEQRRTNSPCQASNGDRHFCGCDRTGVVQCKGGKWTEVQDCGKNSCHGGIEGGAKC** | | | |
| *Penicillium lanosocoeruleum* ATCC 48919 | 385694\|fgenesh1_kg.7_*_91_*_Locus9265v1rpkm1.74 | +0.8 / +1.1 | -0.32 / -0.90 |
| MKFTAMLFTLMAATAVSASVLETR**DGCGSAYGPDQRRTNSPCQSSNGNKQYCGCDRSGIVQCKGGKWTEVQDCGNSPCHGGKEGGALC** | | | |
| *Penicillium polonicum* IBT 4502 | 5065\|PENPOL_c002G02444T0 | +0.8 / +0.1 | -0.30 / -0.61 |
| MKVTAILFTLMAATAVSASVLDQR**DACGSGYDPAQRRTNSPCKSSNGDRHFCGCDRTGIVECKGGKWTEIQDCGRNSCHGGTEGGAKC** | | | |
| *Penicillium swiecickii* 182 6C1 | 30456\|CE30455_51380 | -0.1 / +1.1 | -0.29 / -0.98 |
| MKFTGIFVTLMAATAVSASVLEAR**DACGAGYDPAQRRTNSPCQASNGDRHFCGCDRTGIVECKGGKWTEIQDCGTNSCSGGIEGGAKC** | | | |
| *Penicillium vulpinum* IBT 29486 | 4057\|PENVUL_c023G02320T0 | -0.1 / -0.2 | -0.29 / -0.20 |
|  |  |  |  |
| **NFAP2-cluster proteins** | | | |
| MHLSTALFSAIALLAATQVIGASVEVPRDVAAIQ**IATSPYYACNCPNNCKHKKGSGCKYHSGPSDKSKVISGKCEWQGGQLNCIAT** | | | |
| *Neosartorya fischeri* NRRL 181 | 5870\|7000001156989678 | -0.2 | -0.93 |
| MLFGATLFSAIALLAANQVVGAAVEVPRSVGAVE**IATSAYYACNCPNNCSHKQGSSCKFYSGPSDNSPVISGKCEYQGSQLNCIAK** | | | |
| *Paecilomyces niveus* CO7 | 79875\|gm1.8851_g | -0.2 | -1.00 |
